# *FLC* genes control flowering time to varying degrees in a *Brassica napus* spring cultivar

**DOI:** 10.1007/s11103-026-01741-7

**Published:** 2026-07-29

**Authors:** Sarah Duveneck, Kea Ille, Siegbert Melzer

**Affiliations:** Plant Developmental Biology and Physiology, Kiel University, Am Botanischen Garten 5, 24118 Kiel, Germany

**Keywords:** *Brassica napus*, *FLOWERING LOCUS C*, Floral transition, CRISPR, Epigenetic regulation, Spring oilseed rape

## Abstract

In the crop *Brassica napus* (oilseed rape), distinct growth types have been established that differ mainly in their vernalization requirement for flowering. The need for vernalization in Arabidopsis is controlled by the expression of the floral repressor *FLOWERING LOCUS C* (*FLC*), which also regulates cold-responsive flowering in *B. napus*. Notably, *FLC* homologs are also retained in spring oilseed rape despite its lack of vernalization requirement. To elucidate the functions of the nine *BnFLC* homologs in a spring type, we generated CRISPR/Cas9 knockout mutants of all homologs in the cultivar Westar. We show that the loss of *BnFLC* genes significantly accelerates flowering, demonstrating that *BnFLC* genes regulate flowering in spring types independently of vernalization. Transcriptomic analyses in leaves revealed distinct expression patterns among the *BnFLC* genes, with some remaining active during floral transition. Finally, no epigenetic regulation of the *BnFLC* homologs associated with flowering time was detected, while *BnFLC.A03b* carried persistent repressive marks and was constitutively silenced. Unexpectedly, additional flowering time regulators, including genes typically active in the shoot apical meristem, were expressed in leaves, with some showing altered expression and chromatin states in the mutant. These findings reveal that *BnFLC* genes are developmentally regulated and directly control flowering in spring oilseed rape, while also modulating the expression of other floral regulators in *B. napus*.

**Supplementary Information:**

The online version contains supplementary material available at 10.1007/s11103-026-01741-7.

## Key message

CRISPR/Cas9 knockout of all nine *BnFLC *homologs reveals that these genes regulate flowering time developmentally in spring oilseed rape while influencing the expression and chromatin states of downstream flowering regulators.

**Supplementary Information:**

The online version contains supplementary material available at 10.1007/s11103-026-01741-7.

## Introduction

Plants have evolved diverse strategies to adapt their life cycles to varying environmental conditions. Among these, flowering time is a key adaptive trait that must be precisely regulated to ensure successful reproduction. The transition from vegetative to reproductive growth at the shoot apical meristem (SAM) is controlled by several environmental cues, including photoperiod and temperature (Andrés and Coupland 2012; Friedman 2020). These regulatory mechanisms are particularly important in oilseed rape (*Brassica napus* L.), a major oil crop cultivated worldwide. *B. napus* is an allotetraploid species that originated from spontaneous hybridization of *Brassica rapa* and *Brassica oleracea* (Chalhoub et al. 2014). Within this species, different growth types have been developed that are distinguished primarily by their vernalization requirement. Winter types are biennial and require prolonged exposure to cold (vernalization) to induce flowering. This ensures synchronized flowering under favorable spring conditions. In contrast, spring types are annuals that do not require vernalization. After spring sowing they initiate flowering and complete their life cycle within the same growing season (Friedt and Snowdon 2009; Leijten et al. 2018).

The vernalization pathway has been extensively studied in the model plant *Arabidopsis thaliana*, which, like oilseed rape, belongs to the Brassicaceae family. In winter annual Arabidopsis accessions the central regulator of this pathway is *FLOWERING LOCUS C* (*FLC*), a MADS-box transcription factor that functions as a floral repressor (Sheldon et al. 1999; Michaels and Amasino 1999). FLC blocks flowering by binding to the promoter of the key flowering regulator *FLOWERING LOCUS T* (*FT*) in leaves, thereby inhibiting its expression. In addition, FLC acts at the SAM where it represses the floral integrator *SUPPRESSOR OF OVEREXPRESSION OF CONSTANS 1* (*SOC1*) and *FLOWERING LOCUS D* (*FD*), preventing floral induction (Searle et al. 2006). Beyond these targets, FLC binds to numerous other flowering time regulators (Deng et al. 2011), including *SQUAMOSA PROMOTER BINDING PROTEIN-LIKE 15* (*SPL15*), which promotes the floral transition at the SAM in short days (Hyun et al. 2016), and *SEPALLATA 3* (*SEP3*), which is involved in flower development and floral organ identity (Pelaz et al. 2000; Smaczniak et al. 2012). FLC also targets floral repressors like *TEMPRANILLO1* (*TEM1*) and *SCHLAFMÜTZE* (*SMZ*) (Deng et al. 2011). Importantly, FLC does not always act alone but can form repressive complexes with other MADS-box transcription factors, including *SHORT VEGETATIVE PHASE* (*SVP*) and *MADS-AFFECTING FLOWERING* (*MAF*) to regulate specific target genes (Gu et al. 2013; Mateos et al. 2015).

*FLC* expression is downregulated during vernalization by epigenetic silencing (Sung and Amasino 2004; Bastow et al. 2004; De Lucia et al. 2008; Angel et al. 2011). Before vernalization, the *FLC* locus has the activating histone marks H3K4me3 and H3K36me3. During prolonged cold exposure, these activating histone marks are progressively replaced by the repressive histone mark H3K27me3. The accumulation of H3K27me3 begins at a nucleation region directly downstream of the transcription start site and increases throughout the cold period. Following the return to warm conditions, H3K27me3 marks spread across the entire *FLC* locus, resulting in stable gene silencing (Angel et al. 2011; Yang et al. 2014). This reduction in *FLC* expression permits the expression of flowering promoting genes like *SOC1* and *FT*, ultimately leading to the transition to flowering (Searle et al. 2006).

In Arabidopsis, variation in *FLC* expression reflects the distinction between summer and winter annual growth habits. Summer annuals, which do not require vernalization, typically exhibit low *FLC* expression due to loss-of-function mutations in *FRIGIDA*, a key activator of *FLC*, or the presence of weak alleles of *FLC* (Johanson et al. 2000; Michaels et al. 2003; Lempe et al. 2005). In oilseed rape, *BnFLC* genes were therefore studied as candidate determinants for growth type differentiation. Nine *FLC* homologs have been identified among growth types (Zou et al. 2012; Song et al. 2020), although not all of them are responsive to cold (Schiessl et al. 2019). Early studies showed that at least five *BnFLC* genes can delay flowering when expressed in Arabidopsis (Tadege et al. 2001). Subsequent analyses revealed growth-type-specific differences in *BnFLC* expression levels, suggesting an important role in determining vernalization requirement. In particular, *BnFLC.A02*, *BnFLC.A03b*, *BnFLC.A10* and *BnFLC.C02* show stronger correlations with growth type and vernalization requirement than other homologs (Hou et al. 2012; Chen et al. 2018; Schiessl et al. 2019; Tudor et al. 2020; Song et al. 2020; Yin et al. 2020; Jones et al. 2024). For *BnFLC.A10*, a transposon insertion in the promoter region leads to increased expression and is predominantly found in winter types (Hou et al. 2012; Song et al. 2020; Yin et al. 2020), whereas a transposon insertion in exon 1 correlates with reduced expression in spring types. Similarly, a transposon insertion in exon 7 of *BnFLC.A02* occurs mainly in spring types (Song et al. 2020; Yin et al. 2020). However, since no single *BnFLC* homolog fully accounts for growth type differences, a gene dosage effect, considering both pre-vernalization levels and cold responsiveness of individual homologs, may provide an additional explanation (Calderwood et al. 2021). Consequently, the precise contribution of each *BnFLC* gene to vernalization requirement and flowering time remains unresolved, especially as *BnFLC* genes are still expressed in spring oilseed rape, which lacks a vernalization requirement. The extent to which *BnFLC* genes influence flowering time independently of cold in spring types remains unclear. Moreover, evidence for epigenetic regulation of *BnFLC* genes is currently limited. Available data indicate that *BnFLC.A03b* and *BnFLC.C02* carry activating H3K4me3 marks before winter, which decline after floral transition in winter type oilseed rape (Lu et al. 2022). While in *B. rapa BrFLC* genes accumulate repressive H3K27me3 marks during vernalization (Akter et al. 2019). Overall, the epigenetic regulation of the *BnFLC* genes in oilseed rape remains largely uncharacterized.

In this study, we analyzed the function of *BnFLC* genes in the spring oilseed rape cultivar Westar by generating knockout mutants for all *BnFLC* genes. We show that loss of *BnFLC* function in Westar caused earlier flowering, with the magnitude of the effect varying among mutants. Transcriptomic analyses of leaves from wild-type and mutant plants further revealed distinct expression patterns of flowering time regulators across developmental stages up to flowering. Moreover, we performed genome-wide profiling of the histone marks H3K4me3 and H3K27me3 to identify epigenetically regulated genes and to determine whether these chromatin landscapes differ between mutant and wild-type plants.

## Materials and methods

### Plant material and growth conditions

The spring *Brassica napus* cv. Westar was used for phenotyping and plant transformation. Phenotyping for flowering time was conducted in a greenhouse under long-day conditions (16 h light/8 h dark) with temperatures ranging from 20 to 28 °C. Flowering time was evaluated for 10–12 plants per genotype. The appearance of the first open flower was defined as the onset of flowering and recorded as days to flowering (DTF). Plants used for transcriptomic, epigenetic and microscopic analyses were grown in climate chambers under long day conditions at a constant temperature of 20 °C.

### Identification of CRISPR/Cas9 target sites for *BnFLC* genes

The *BnFLC* genes in the reference genome of Westar (Song et al. 2020) were identified by using the *AtFLC* coding sequence (CDS) as a query for a BLASTN search. The CDS of the *BnFLC* genes were manually adapted (corresponding IDs are provided in Table S1). The sequence-specific target sites were designed using CRISPR-P 2.0 (Liu et al. 2017) and were further checked for potential off-target sites within the Westar reference genome. The target sites and oligonucleotides are presented in Table S2 and were ordered from Eurofins Genomics (Munich, Germany).

### Vector construction and plant transformation

The “MoClo CRISPR/Cas Toolkit for Plants” (Hahn et al. 2020) was used for the construction of the CRISPR/Cas9 transformation vector. Additionally, the plasmids pICH47751 (Addgene plasmid #48002), pICH41766 (Addgene plasmid #48018) and pAGM65881 (Addgene plasmid #153215) were used, which were a gift from Sylvestre Marillonnet (IPB Halle, Germany). The final CRISPR/Cas9 vector contained a polycistronic tRNA-sgRNA transcriptional unit driven by the RNA polymerase III promoter AtU6-26. It included six target sites designed to target all nine *BnFLC* genes in Westar.

Vector assembly was performed according to the corresponding protocol of the kit. Briefly, the target sites were cloned into suitable L0-vectors and then subcloned into the L1-vector pICH47751. Finally, the complete transcriptional unit was assembled into the CRISPR/Cas9 vector pAGM65881. The resulting CRISPR/Cas9 vector was verified by restriction digestion and whole-plasmid sequencing using Plasmid EZ (Genewiz, Leipzig, Germany). The CRISPR/Cas9 vector was transformed into the *Agrobacterium tumefaciens* strain GV3101::pMP90 for plant transformation. An *Agrobacterium*-mediated hypocotyl transformation of Westar was performed according to the protocol of Ille and Melzer (2025). This protocol is based on the co-transformation of hypocotyls with one *Agrobacterium* culture carrying the CRISPR/Cas9 vector and a second *Agrobacterium* culture carrying a vector with the 35S::*BvWUS* gene.

### Genotyping of regenerated plants

Regenerated and rooted plants were transferred to soil and were screened for the presence of transgenes from both vectors. Genomic DNA was isolated using a slightly modified protocol based on Dellaporta et al. (1983). Specific primers targeting either the kanamycin resistance gene of the CRISPR/Cas9 vector or the *BvWUS* gene were used for analysis. Plants positive for the CRISPR/Cas9 vector were further analyzed for editing at the target sites using Amplicon EZ (Genewiz, Leipzig, Germany). Amplicons spanning the target sites, with a maximum size of 500 bp, were generated by PCR using the Q5 polymerase (NEB, Frankfurt, Germany). As the genes have a high sequence similarity, we amplified *BnFLC.A02/C02*, *BnFLC.A03a.1/A03a.2*, *BnFLC.A03b/C03b* and *BnFLC.C09a/C09b/A10* together and were able to distinguish them later based on SNPs. PCR products from up to three plants were pooled, as primers with specific barcodes were employed for each plant. After sequencing, the paired-end reads of the pooled sequences were first merged using fastp with standard settings for paired-end sequences and the --dont_eval_duplication option. Reads were then demultiplexed based on the assigned barcodes using a customized grep command. The resulting sequences for each plant were analyzed by CRISPResso2 (Clement et al. 2019) in the CRISPRessoPooled mixed mode using the following parameters: --min_reads_to_use_region 100 --demultiplex_only_at_amplicons --bam_output. Additionally, amplicons were manually examined using IGV (Robinson et al. 2011). The progenies of the primary transformants were further analyzed and selected to get plants with a stable genotype. The genotypes of all plants used in this study are presented in Table S3 and S4.

### Gene expression analysis

The Westar wild type and the mutant *flc-W6-1* were used for transcriptomic analysis. Plants were grown in a climate chamber, and samples were collected every week after sowing until flowering. To obtain seedlings for sampling at week 0, seeds were germinated on filter paper in the climate chamber. After three days, whole seedlings were harvested, with five seedlings pooled per replicate. For all subsequent time points, leaf samples from three plants were pooled per replicate. The specific leaf types sampled at each time point are listed in Table 1.

Total RNA was isolated as described by Melzer et al. (1990). Library preparation and RNA sequencing, with two replicates per time point, were carried out at Novogene GmbH (Munich, Germany). Paired-end reads (150 bp) were generated using Illumina NovaSeq X Plus system. The raw fastq files were processed using nf-core/rnaseq (v3.18.0; doi: 10.5281/zenodo.1400710) of the nf-core collection of workflows (Ewels et al. 2020). Reads were aligned to the Westar reference genome using STAR (Dobin et al. 2013), and transcript quantification was conducted with Salmon (Patro et al. 2017). The reference file for the transcripts was manually adjusted for the *BnFLC* genes. Visualization was based on transcript per million (TPM) values. Differential gene expression analysis was performed on raw counts using DeSeq2 (Love et al. 2014). The model accounted for both genotypes and time points using the formula: design = ~ genotype + time + genotype:time. Genes with an absolute log2 fold change ≥ 2 and an adjusted p-value ≤ 0.01 were considered as differentially expressed genes (DEGs).

### CUT&Tag for genome-wide profiling of histone marks

Cleavage Under Targets and Tagmentation (CUT&Tag; Kaya-Okur et al. 2019) was used to analyze genome-wide profiles of the histone marks H3K27me3 and H3K4me3 in the Westar wild type and the mutant *flc-W6-1*. Leaf samples were collected at different time points before flowering. Sampling was conducted at week 2, 5 and 6 for Westar, and at week 2 and 5 for *flc-W6-1*. The sampled leaf types are listed in Table 1. For each time point, two replicates per histone mark were sampled and each replicate consisted of pooled material from three plants. As negative control an IgG sample was used. From 0.2 g of leaves nuclei were isolated following a modified protocol from Fu et al. (2024). Leaf samples with two steel beads were frozen in liquid nitrogen and ground twice for 15 s at 55 Hz with a homogenizer (Servicebio, China). Afterwards 800 µl Honda buffer were added and incubated for 20 min on ice. The sample was filtered through a 30 μm CellStrainer (CellTrics) into a new 2 ml tube. An additional 600 µl Honda buffer were used to wash the first tube and the filter. The filtrate was centrifuged for 2 min at 400 x g, and the flowthrough was centrifuged at 4000 x g for 15 min at 4 °C. The pellet was resuspended in 1 ml Honda buffer and centrifuged again at 4000 x g for 15 min at 4 °C. The resulting pellet (contained around 60000 nuclei) was used as input for the CUTANA CUT&Tag Kit (Epicypher, Durham, USA) and resuspended in 110 µl of the Nuclear Extraction Buffer. All subsequent steps were carried out according to manufacturer’s protocol. As antibodies Anti-H3K27me3 (Epicypher, #13–0055), Anti-H3K4me3 (Epicypher, #13–0060), IgG control (Epicypher, #13–0042) and Anti rabbit secondary antibody (Epicypher, #13–0047) were used. Paired-end sequencing (150 bp) of the library was performed at Novogene GmbH (Munich, Germany) using the NovaSeq X Plus system, generating approximately 3 Gb of raw data per sample. The raw fastq files were processed using nf-core/cutundrun (v3.2.2, doi: 10.5281/zenodo.5653535) from the nf-core collection of workflows (Ewels et al. 2020). Reads were aligned with Bowtie2 (Langmead and Salzberg 2012) and MACS2 (Zhang et al. 2008) was used for peak calling. Normalization was performed using the “CPM” mode. Differentially enriched peaks were identified by using DeSeq2 (Love et al. 2014), using raw counts per peak region as input. Peaks with an absolute log2 fold change ≥ 1 and an adjusted p-value ≤ 0.01 were considered as differently enriched.

### Microscopy

For microscopic analysis of SAM development, the Westar wild type and the mutant *flc-W6-1* were grown in a climate chamber under the same conditions as used for the transcriptomic and epigenetic analysis. Apices were manually dissected at the time points week 2, 3 and 4 for both genotypes. Samples were fixed in 4 % paraformaldehyde overnight. Then the samples were dehydrated through a graded ethanol series and embedded in Technovit 7100 (Kulzer, Germany) according to the manufacturer’s protocol. Embedded apices were sectioned at 5 μm using a Leica rotary microtome and stained with 0.5 % toluidine blue.

**Table 1:** Sample types used for the analysis of gene expression and histone marks at each weekly time point

Sampling time point	Westar	*flc-W6-1*
W0	Whole seedling	Whole seedling
W1	Cotyledons	Cotyledons
W2	Newest leaf	Newest leaf
W3	Newest leaf	Newest leaf
W4	Newest leaf	Newest leaf
W5	Newest leaf	Newest cauline leaf
W6	Newest cauline leaf	Not included

## Results

### Targeting multiple *BnFLC* genes with CRISPR/Cas9 in Westar

**Fig. 1:**
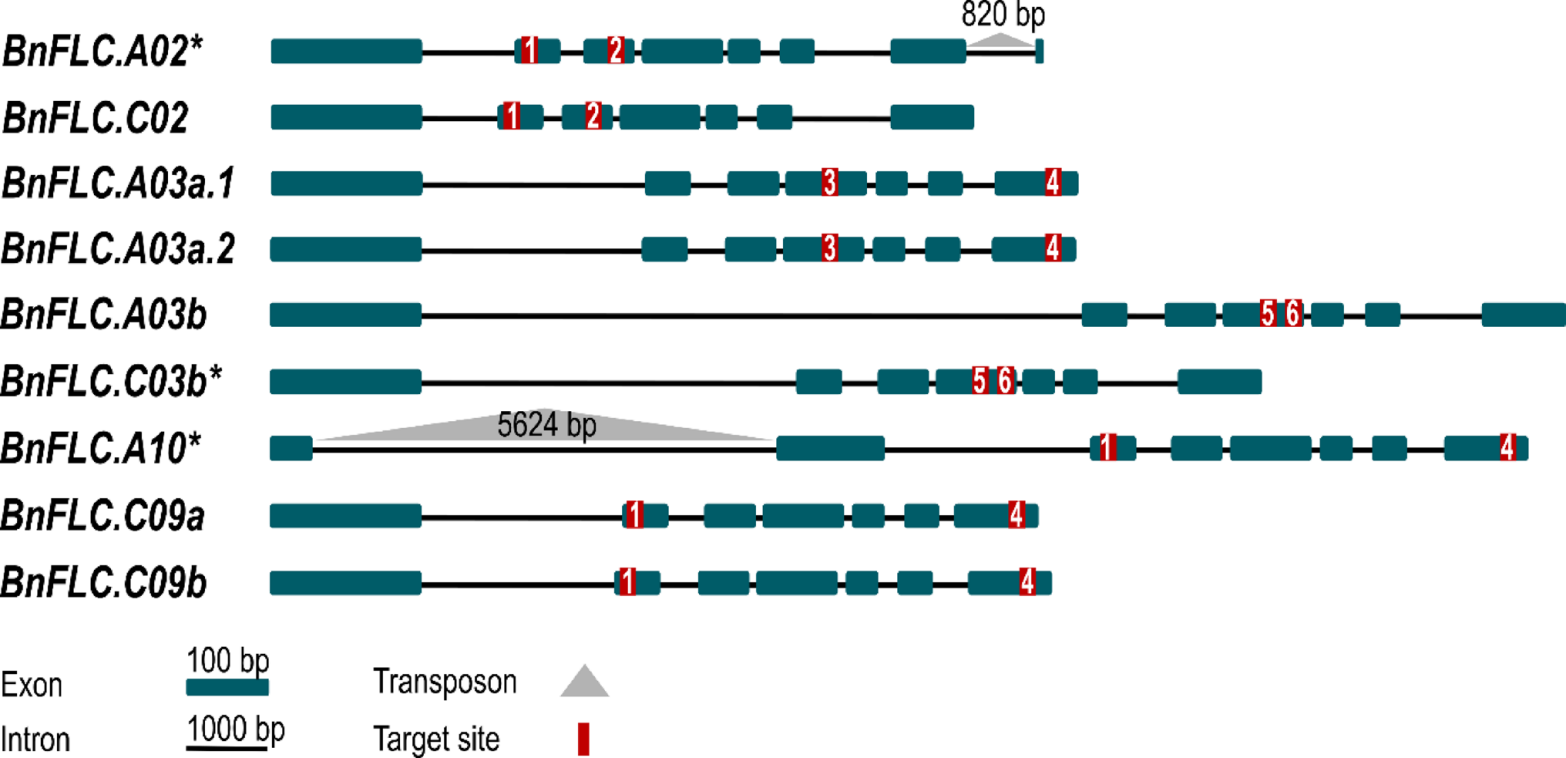
Gene structures of the *BnFLC* genes in Westar, from the translational start to stop site. Red boxes indicate the positions of the selected CRISPR/Cas9 target sites. Grey triangles denote transposon insertions. Non-functional genes are marked with an asterisk (*)

The *BnFLC* genes are known to influence the vernalization requirement of *B. napus* through differences in their expression levels and are thought to contribute to the differentiation of growth types (Schiessl et al. 2019; Song et al. 2020; Yin et al. 2020; Calderwood et al. 2021). Although FLC functions as a floral repressor in the vernalization pathway from Arabidopsis, *BnFLC* genes are also expressed in spring oilseed rape, which does not require vernalization. To assess their role in flowering time control in a spring background, we generated knockout mutants for the *BnFLC* genes in the spring cultivar Westar. Homologs of *AtFLC* were identified in the Westar reference genome (Song et al. 2020), revealing nine *BnFLC* genes (see Table S1), which were named according to Calderwood et al. (2021). In contrast to previous reports for Westar (Schiessl et al. 2019; Calderwood et al. 2021; Jones et al. 2024), we identified only a single *BnFLC* gene on chromosome C03, whereas three were detected on chromosome A03. This discrepancy is likely attributable to differences in the reference genomes used, as previous studies employed the Darmor-*bzh* reference genome (Chalhoub et al. 2014), whereas we used the Westar reference genome (Song et al. 2020). Sequence analysis indicated that in Westar, *BnFLC.A03a* is duplicated, while *BnFLC.C03a* is absent. The duplicated *BnFLC.A03a* genes are identical except for a 26 bp insertion/deletion (InDel) in the first intron, which was confirmed by PCR (Fig. S1). Previously reported transposon insertions in *BnFLC.A02* and *BnFLC.A10*, which abolish gene expression (Song et al. 2020; Yin et al. 2020), were also identified. In addition, *BnFLC.C03b* is presumed to be a pseudogene, as it lacked detectable expression in earlier studies, and contains a premature stop codon in exon 2 (Zou et al. 2012). Accordingly, *BnFLC.A02*, *BnFLC.A10*, and *BnFLC.C03b* are likely non-functional and are hereafter denoted with an asterisk (*). As the *BnFLC* genes show a high degree of sequence similarity, conserved regions were identified that allowed the use of shared CRISPR/Cas9 target sites across multiple genes. Accordingly, six target sites were designed, two for each gene, as shown on the gene structures in Fig. 1. The exact target site sequences are listed in Table S2. All target sites were assembled into the multiplex CRISPR/Cas9 vector pAGM65881 (Grützner et al. 2021) which harbors an intronized Cas9 gene driven by the RPS5A promoter from Arabidopsis.

### Generation of CRISPR/Cas9 mutants with varying mutations

*Agrobacterium*-mediated transformation of hypocotyl explants was performed following the protocol of Ille and Melzer (2025), which is based on the co-transformation of a 35S::*BvWUS* transgene to enhance regeneration efficiency in oilseed rape. In total, 442 hypocotyl segments from Westar wild-type plants were transformed, resulting in the regeneration of 15 kanamycin resistant plants, which were subsequently transferred to soil. Eight plants carried the CRISPR T-DNA and were analyzed for gene editing by amplicon sequencing. The targeted regions of *BnFLC.A03a.1* and *BnFLC.A03a.2* were additionally examined by Sanger sequencing, as they could not be distinguished based on SNPs. Therefore, gene specific amplification was performed using primers spanning the InDel region. All eight analyzed plants contained mutations in the *BnFLC* genes, although the number and combination of edited alleles varied among transformants. Biallelic mutations were identified in at least two genes per primary transformant, with the highest number of six biallelic mutations in the T_0_ plant *flc-W7* (Table S3).

### *BnFLC* knockouts accelerate flowering in Westar

**Fig. 2:**
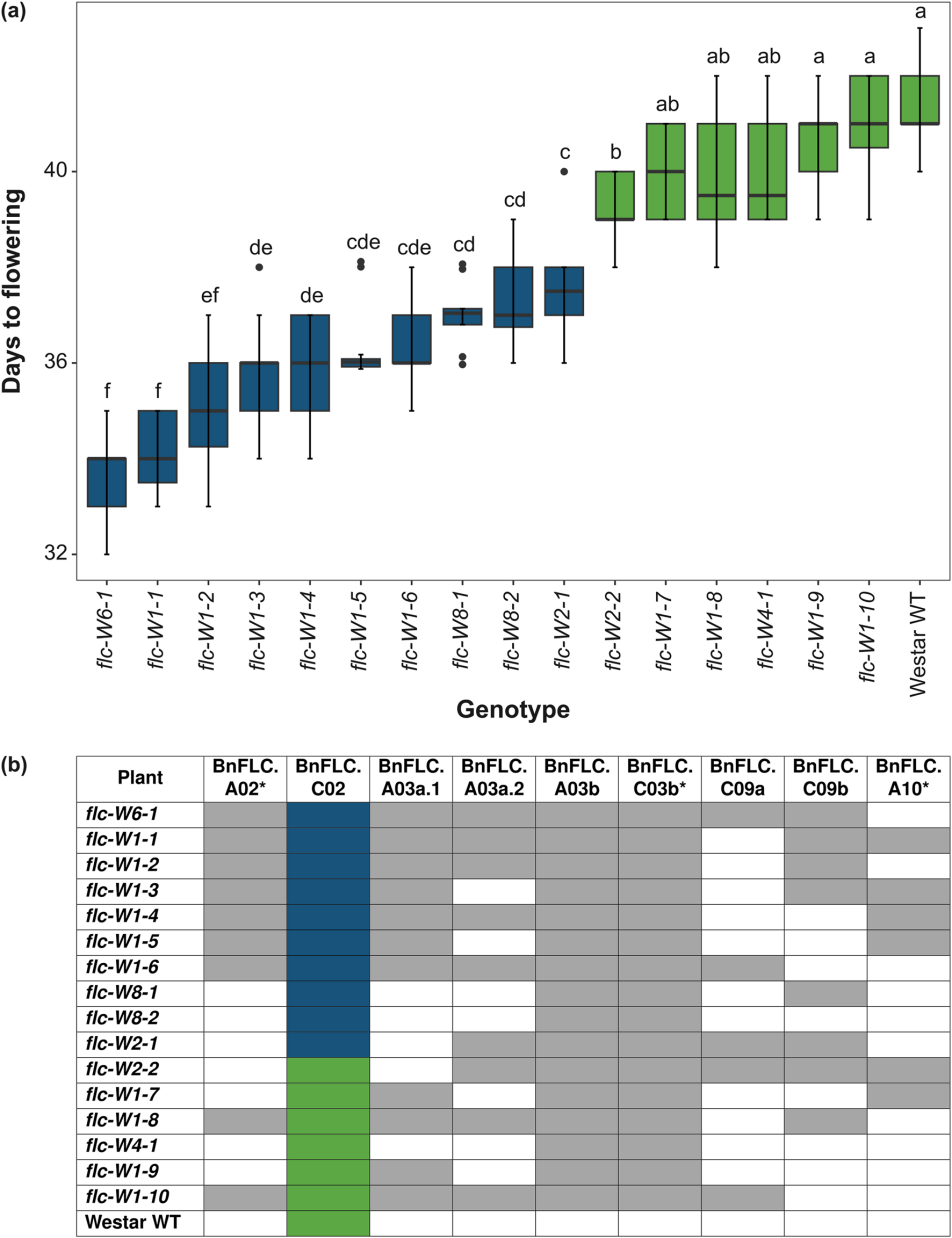
Phenotypic and genotypic analysis of T_2_ and T_3_ plants of *BnFLC* mutants in Westar. **a** Flowering time of *BnFLC* mutants grown in the greenhouse. Plants mutated for *BnFLC.C02* are shown in blue, while plants wild type for *BnFLC.C02* are shown in green. Genotypes are ordered by DTF. Differences in flowering time were assessed by one-way analysis of variance (ANOVA) followed by Tukey’s multiple-comparison test. Different letters indicate significant differences (*p* < 0.01). **b** Genotypes of mutants shown in (a). Genes colored in gray or blue are mutated, whereas genes shown in white or green are wild type. Non-functional genes are marked with an asterisk (*)

**Fig. 3:**
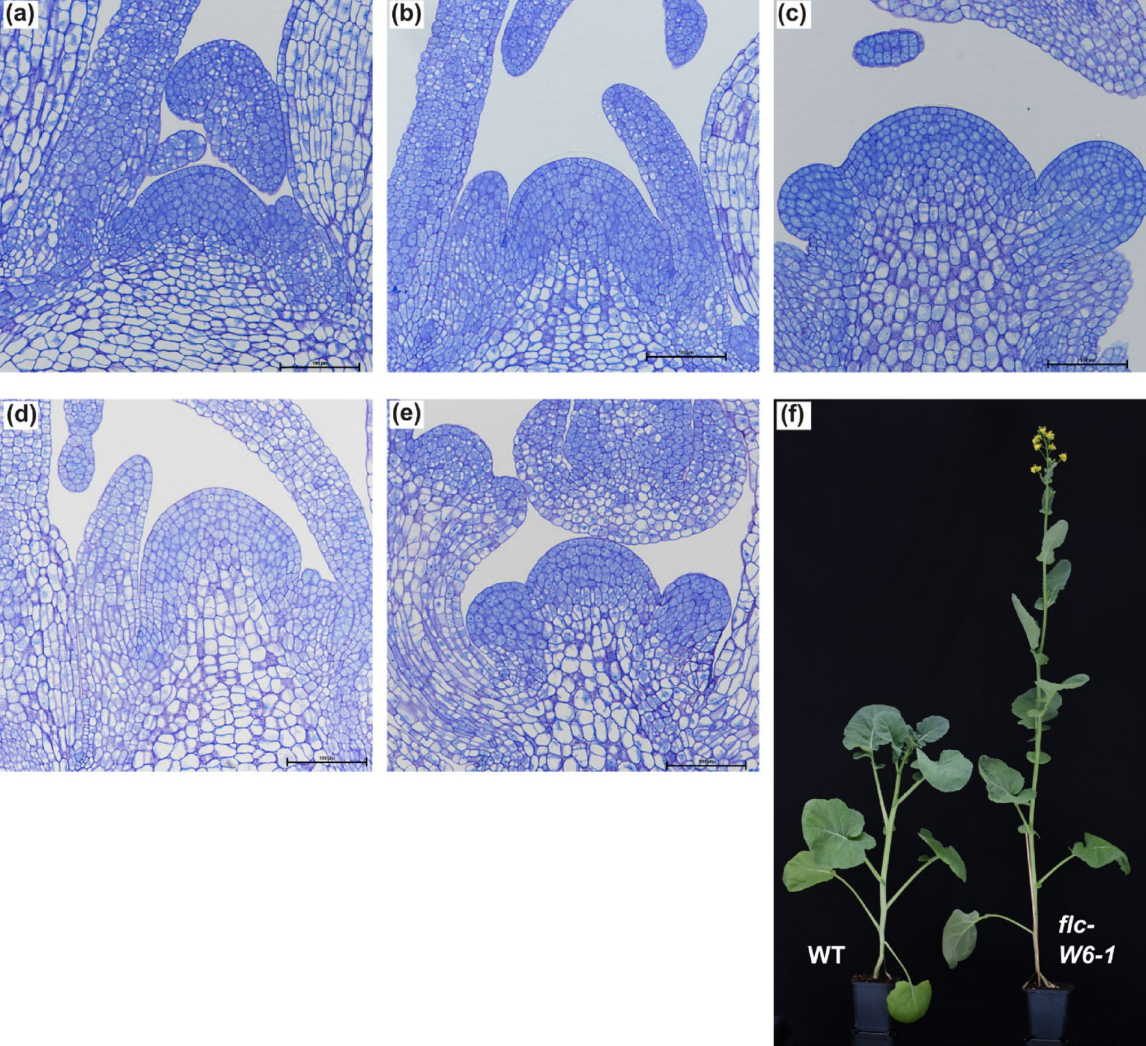
Comparison Westar wild type and the mutant *flc-W6-1*. **a–c** SAMs of Westar at 2 (a), 3 (b) and 4 (c) weeks after sowing. **d–e** SAM of the mutant *flc-W6-1* at 2 (d) and 3 (e) weeks after sowing. Black bars represent 100 μm. **f** Phenotype in week 5 of Westar wild type and a *flc-W6-1* mutant, which flowered one week earlier

**Fig. 4:**
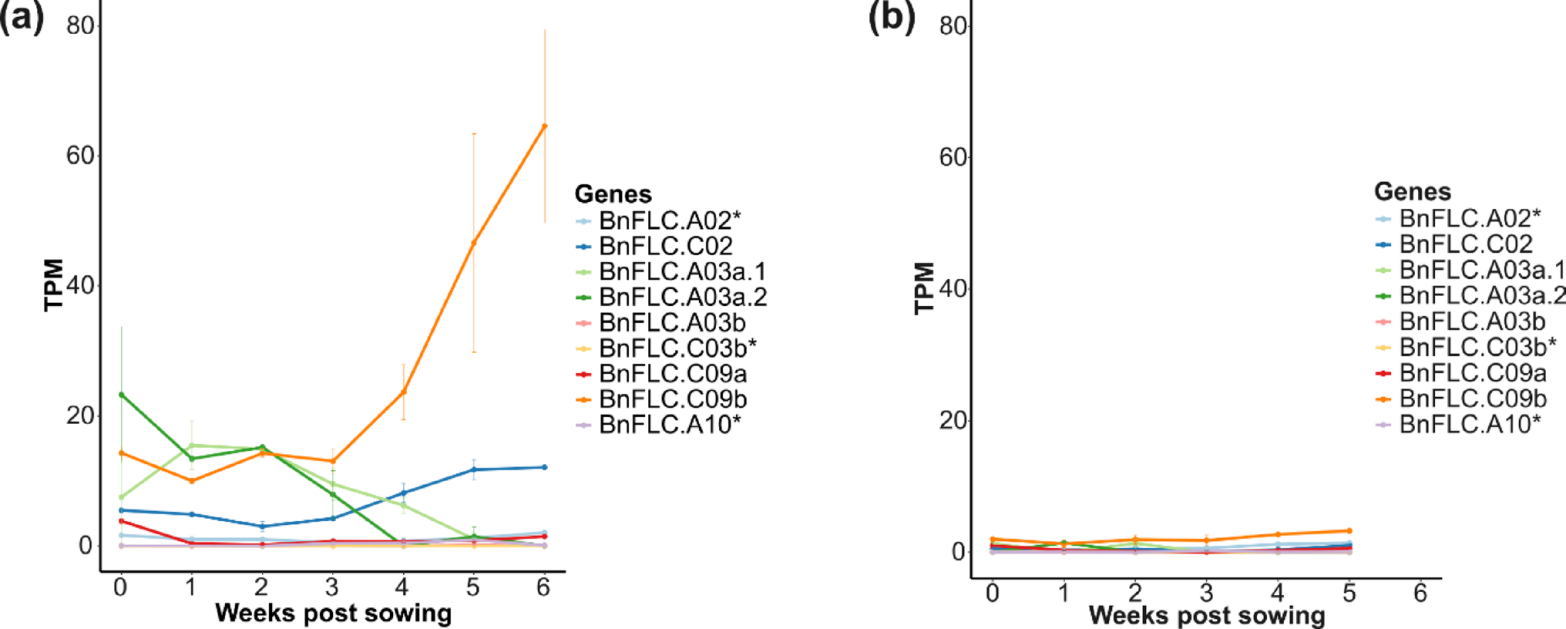
Expression profiles of *BnFLC* genes in the leaves of Westar wild type **a** and the mutant *flc-W6-1* **b** during development until flowering. Gene expression was measured weekly from week 1 (W1) to week 6 (W6), W0 represents the stage of 3-day-old seedlings. Expression values are shown as TPM ± SD

To assess the impact of different combinations of mutated and non-mutated *BnFLC* genes on flowering time in Westar (hereafter referred to as wild type), edited lines with stable allele combinations were selected (Table S4). Among these the line *flc-W6-1* was of particular interest, as it carried mutations in eight out of nine *BnFLC* genes, except for *BnFLC.A10*. We considered this genotype functionally equivalent to a full knockout, since *BnFLC.A10* is not expressed in Westar as reported previously (Song et al. 2020; Yin et al. 2020) and confirmed here by transcriptome analysis (Fig. 4a). Under summer greenhouse conditions, *flc-W6-1* flowered eight days earlier than the wild type (see Figs. 2a, b and 3f). The flowering behavior of *flc-W6-1* was further evaluated under additional conditions. In the greenhouse during winter, flowering was delayed in both wild-type and mutant plants compared with summer greenhouse conditions. However, *flc-W6-1* still flowered 14 days earlier than the wild type. Similarly, under LD conditions at 20 °C in the climate chamber, the mutant flowered 11 days earlier than the wild type, although the absolute time to flowering was shorter than under winter greenhouse conditions (see Table S5).

Furthermore, we assessed the flowering time of mutants carrying different combinations of edited *BnFLC* genes under summer greenhouse conditions (Fig. 2a, b). A continuous distribution of flowering times was observed across the genotypes, suggesting largely additive effects on flowering earliness with increasing numbers of mutated genes. Notably, the earliest flowering consistently occurred in genotypes in which *BnFLC.C02* was mutated. Apart from *flc-W2-2*, plants retaining the wild-type *BnFLC.C02* alleles did not flower significantly earlier than the wild type. In contrast, all plants carrying knockout alleles of *BnFLC.C02* flowered significantly earlier than the wild type, although they showed greater variation in flowering time. Nevertheless, most of these lines still flowered significantly later than *flc-W6-1*, highlighting that flowering time is influenced not only by the presence of *BnFLC.C02* mutations but also by the total number of mutated *BnFLC* genes.

Among the remaining *BnFLC* genes, *BnFLC.C09b* emerged as a candidate contributing to variation in flowering time. In *flc-W1-10*, which retains wild-type alleles of *BnFLC.C02* and *BnFLC.C09b* but carries mutations in most other *BnFLC* genes, flowering time was similar to that of the wild type. A potential effect of *BnFLC.C09b* became apparent when comparing *flc-W6-1* and *flc-W1-6*, which differed significantly in flowering time while differing only at *BnFLC.C09b*. However, this effect appeared less pronounced in genotypes carrying a higher number of wild-type *BnFLC* alleles. For instance, *flc-W8-1* and *flc-W8-2* differed solely at *BnFLC.C09b* but showed no detectable difference in flowering time, indicating that the remaining expressed wild-type genes in *flc-W8-1* and *flc-W8-2* (*BnFLC.A03a.1* and *BnFLC.A03a.2*) compensated the loss of *BnFLC.C09b* in *flc-W8-1*.

To investigate the effect of *BnFLC* gene knockouts on floral transition, SAMs from wild-type and *flc-W6-1* plants were examined at different developmental stages (Fig. 3). In wild-type plants, the SAMs were still in the vegetative stage in week 2 (Fig. 3a). The floral transition began at week 3, as indicated by the emergence of a dome-shaped meristem (Fig. 3b). By week 4, the inflorescence meristem and floral meristems were clearly visible (Fig. 3c). In contrast, *flc-W6-1* plants developed a dome-shaped meristem as early as week 2 (Fig. 3d), indicating an accelerated floral transition in this mutant. This observation is consistent with the approximately one- to two-week earlier flowering observed in *flc-W6-1* plants under different conditions (Table S5).

### Loss of *BnFLC* function activates flowering time genes earlier

The earlier flowering observed in *BnFLC* mutants indicates potential changes in the expression of downstream floral regulators. Examining transcript levels and histone modifications provides insight into how *BnFLC* genes shape flowering time in spring oilseed rape. For transcriptomic analyses, leaf samples were collected weekly, from week 1 to week 6. In *flc-W6-1* sampling was limited to week 5 as flowering commenced at that stage. Week 0 corresponds to 3-day-old seedlings. The exact tissues sampled at each time point are listed in Table 1.

We first examined the expression profiles of *BnFLC* genes in the wild type throughout development until the onset of flowering (Fig. 4a). Of the nine *BnFLC* genes, only four were expressed in leaves. Among these, *BnFLC.A03a.1* and *BnFLC.A03a.2* were fully downregulated during development in the absence of cold treatment. In contrast, the other two expressed genes, *BnFLC.C02* and *BnFLC.C09b*, showed increased expression levels after week 3. Notably, *BnFLC.C09b* was strongly upregulated, reaching nearly threefold higher expression levels at week 6 compared to week 3. In the *flc-W6-1* mutant, almost no *BnFLC* transcripts were detected (Fig. 4b).

To identify genes that may be directly affected by the loss of BnFLC function, we searched for DEGs with distinct temporal expression patterns in leaf transcriptomes in wild-type plants and *flc-W6-1* mutants. Focusing on flowering-related DEGs, we found that *BnSEP1*, *BnSEP2* and *BnSEP3* genes were unexpectedly expressed in leaves and exhibited the most pronounced differences in their expression profiles. The *SEP* genes are known floral organ identity genes in Arabidopsis and are part of the extended ABCE model of flower development (Ditta et al. 2004). Expression profiles of all *BnSEP* homologs in wild-type and *flc-W6-1* plants are shown in Fig. S2. All four *BnSEP1* genes showed increased expression levels in *flc-W6-1* after week 3, whereas in in the wild type, this upregulation occurred only after week 5 (Fig. S2a, b). A similar pattern was observed for *BnSEP2.C01*, *BnSEP2.C05a* and *BnSEP3.A09*, while *BnSEP2.A01* was already upregulated in the mutant after week 2 (Fig. S2c, d). The remaining *BnSEP3* homologs exhibited a later upregulation after week 4 in the mutant, whereas in wild-type plants little to no increase in expression levels was observed (Fig. S2e, f).

By examining genes that were significantly upregulated during the floral transition in wild-type leaves and correspondingly one week earlier in the mutant, we found that some of the *BnSOC1* genes showed earlier upregulation in *flc-W6-1* although their expression levels did not reach those observed in wild-type plants (Fig. S3a, b). In Arabidopsis *SOC1* acts as a floral integrator that is upregulated during floral transition at the SAM and is a direct target of FLC (Borner et al. 2000; Searle et al. 2006). Since *FT* is also directly affected by FLC in Arabidopsis leaves (Searle et al. 2006) we next analyzed the expression of *BnFT* genes (Fig. S3c, d). In the mutant, *BnFT* expression increased after week 3 whereas in the wild-type plants this increase occurred after week 4. Notably, high *BnFT* transcript levels were detected in cauline leaves of *flc-W6-1* at flowering in week 5, while the corresponding wild-type samples at week 6 showed substantially lower expression. Among the *BnFT* homologs, *BnFT.A02* displayed the highest expression. In addition, some *BnFUL* genes exhibited a slightly earlier upregulation in the mutant, particularly *BnFUL.C07* (Fig. S3e, f). *FUL* functions as a floral integrator at the SAM and is also activated in Arabidopsis leaves by FT (Teper-Bamnolker and Samach 2005). The homologs of other flowering time regulators that act at the Arabidopsis SAM during floral transition, such as *SPL15* (Hyun et al. 2016) and *AP1* (Liljegren et al. 1999), displayed heterogeneous and generally lower expression among their homologs in wild-type leaves (Fig. S4a-d). *BnSPL15.C04* showed a slight increase in transcript levels in the wild type that was not observed to the same extent in the mutant, whereas other homologs were expressed at lower levels. Of the six *BnAP1* homologs, only two were expressed in leaves. *BnAP1.C02* maintained relatively constant expression throughout development in both genotypes, whereas *BnAP1.A02* underwent a steady increase over time. In contrast, none of the *BnLFY* homologs, a key regulator of floral meristem identity together with *AP1* in Arabidopsis (Weigel et al. 1992), were expressed in leaves (Fig. S4e, f).

Apart from flowering time regulators, we identified several genes involved in hormone biosynthesis and signaling that were differently expressed between mutant and wild-type plants. Notably, two *GIBBERELLIN 20-OXIDASE 3* (*BnGA20OX3*) homologs (*BnGA20OX3.A03* and *BnGA20OX3.nn*), which catalyze the final biochemical step in the biosynthesis of bioactive GAs (Coles et al. 1999; Plackett et al. 2012), showed a sharp upregulation in both genotypes one week after sowing. Transcript levels were approximately twofold higher in the mutant compared with the wild type at this stage but declined in both genotypes in subsequent weeks (Fig. S5a, b). In addition, the *HOMOLOG OF BEE2 INTERACTING WITH IBH1* (*HBI1*) and *BR-ENHANCED EXPRESSION 2 (BEE2*) genes were differently expressed in the mutant compared to wild type (Fig. S5c-f). HBI1 and BEE2 are transcription factors and act as positive regulators of brassinosteroid (BR) signalling (Friedrichsen et al. 2002; Cai et al. 2021). BR-deficient plants show late flowering phenotypes in Arabidopsis (Li et al. 2010). In our study, all *BnHBI1* genes as well as *BnBEE2.A08* and *BnBEE2.C03* were upregulated in week 1 compared to the wild type.

### Epigenetic modifications associated with flowering in wild-type and mutant plants

In Arabidopsis the epigenetic regulation of *FLC* in response to cold exposure has been well characterized, with the accumulation of repressive H3K27me3 histone marks at the *FLC* locus leading to stable transcriptional silencing (Sung and Amasino 2004; Bastow et al. 2004; Schubert et al. 2006). To determine whether *BnFLC* genes in Westar are similarly regulated, and whether other flowering regulators might show different chromatin states between wild-type and *flc-W6-1* mutant plants, we analyzed the distribution of the repressive histone mark H3K27me3 and the activating histone mark H3K4me3 in the youngest visible leaves. Samples were collected at week 2 and week 5 for both genotypes. For the wild type, week 6 was additionally analyzed to match the developmental stage of week 5 in the mutant. Genome-wide profiling revealed the expected distribution patterns for both histone marks. H3K27me3 was broadly distributed across gene bodies, whereas H3K4me3 was enriched around transcription start sites (representative heatmaps are displayed in Fig. S6a). Biological replicates showed high consistency across all time points for both marks, with correlation coefficients exceeding 0.9 (Fig. S6b).

We first examined the histone marks of the *BnFLC* genes. Strong repressive H3K27me3 marks were detected at the *BnFLC.A03b* locus, which was identified as non-expressed in the transcriptomic analysis (Fig. S7a-b). In contrast, the remaining *BnFLC* loci showed no or only weak (*BnFLC.C02*) H3K27me3 enrichment. Activating H3K4me3 marks were absent or present only at very low levels (*BnFLC.A03b* and *BnFLC.C02*) at all *BnFLC* genes. The histone marks for the remaining *BnFLC* loci are shown in Fig. S7c-l, excluding the non-functional genes *BnFLC.A02*, *BnFLC.A10*,* and BnFLC.C03b*.

**Fig. 5:**
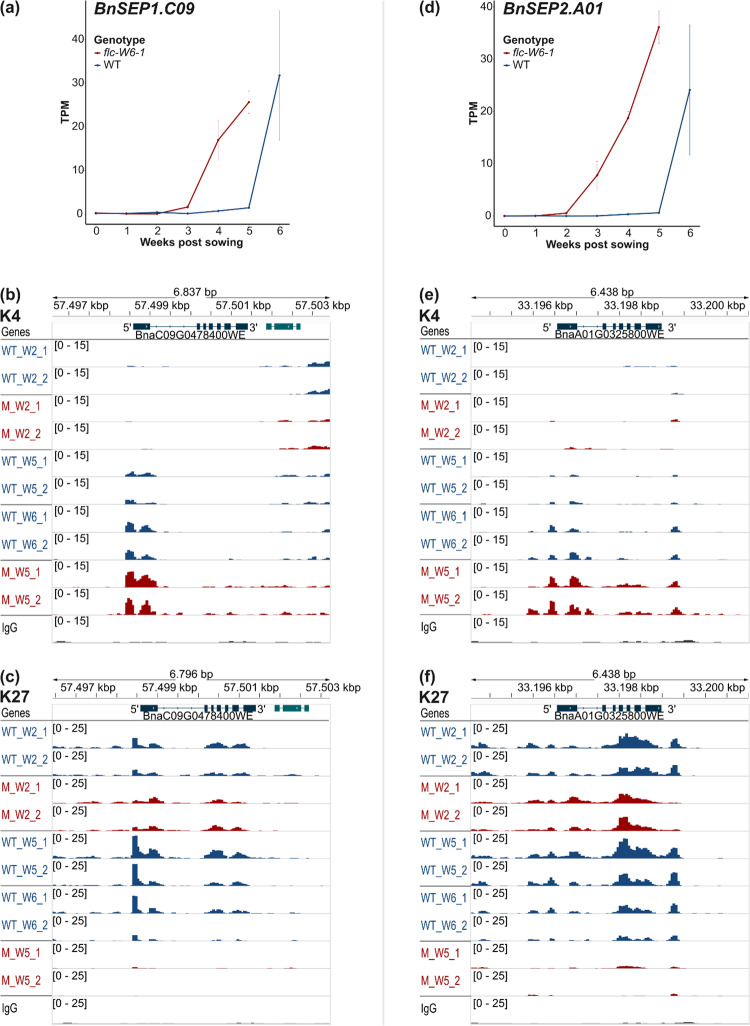
Expression profiles and histone marks of *BnSEP1.C09* and *BnSEP2.A01* in Westar wild type and the mutant *flc-W6-1*. **a**–**c** Expression profile **a**, profiles of H3K4me3 marks **b**, and profiles of H3K27me3 marks **c** of *BnSEP1.C09*. **d**–**f** Expression profile and histone mark profiles of *BnSEP2.A01* are presented in the same way. The profiles of the mutant *flc-W6-1* are shown in red and of Westar in blue. Expression values are shown as TPM ± SD. For histone marks two replicates per time point are displayed. IgG is shown as negative control

We next assessed whether histone mark enrichment differed between wild-type and mutant plants at individual time points, as well as how these marks changed over time within each genotype. Histone mark profiles are shown for one homolog and described for the remaining homologs. Across multiple comparisons, *BnSEP1* and *BnSEP2* genes showed differential enrichment of histone marks. As these genes were also identified in the transcriptomic analysis, we examined them in greater detail. For *BnSEP1.C09* (Fig. 5a-c) and *BnSEP2.A01* (Fig. 5d-f), the *flc-W6-1* mutant exhibited an earlier and stronger increase of H3K4me3 marks between weeks 2 and 5. Conversely, H3K27me3 levels decreased from early to late stages, with this reduction occurring earlier and reaching lower levels in the mutant. This chromatin signature is consistent with the earlier transcriptional activation of these genes. Similar patterns were observed for all *BnSEP1* homologs and *BnSEP2.C01*. In contrast, *BnSEP2.C05a* and *BnSEP2.C05b* retained H3K27me3 marks in both genotypes at all time points, whereas no H3K4me3 marks were detected.

The *BnFUL* genes showed dynamic epigenetic regulation, exemplified here by *BnFUL.C07* (Fig. S8a-c). Repressive H3K27me3 marks were detected at this locus at week 2 and week 5 in the wild type, and to a lesser extent in week 2 in the mutant. In cauline leaves of both genotypes, H3K27me3 was strongly reduced and accompanied by the appearance of activating H3K4me3 marks, which were absent at week 2. This shift in histone modification patterns is consistent with the developmental progression of both genotypes and their expression profiles. Similar dynamics were observed for *BnFUL.A03*, *BnFUL.A09* and *BnFUL.C09*. For *BnFUL.A02* and *BnFUL.C02* H3K4me3 marks were also present, but H3K27me3 enrichment was retained, indicating a more complex chromatin state.

Some *BnSOC1* homologs (*BnSOC1.A03*, *BnSOC1.A04* and *BnSOC1.A05*) exhibited dynamic changes in histone modifications during development, although no significant differences between genotypes were detected. Low levels of H3K4me3 were detected at week 2 in both genotypes and increased by week 5, whereas H3K27me3 levels were low at early stages and disappeared completely at later stages. Interestingly, H3K4me3 marks appeared more prominent at week 5 in the wild type than in *flc-W6-1* (Fig. S8d-f). A similar trend, but with lower levels of these histone marks, occurred for *BnSOC1.C03* and *BnSOC1.C04a*. In contrast, the non-expressed homolog *BnSOC1.C04b* harbored exclusive enrichment of H3K27me3 marks. Epigenetic regulation was also evident for *BnSPL15* genes. *BnSPL15.C04*, which was constitutively expressed throughout development, carried only activating H3K4me3 marks in both genotypes (Fig. S9a-c), while *BnSPL15.A04* showed weaker H3K4me3 enrichment. The other two homologs lacked detectable histone marks. In contrast all four *BnAP1* homologs that were not expressed in leaves (*BnAP1.A07a*, *BnAP1.A07b*, *BnAP1.C06a* and *BnAP1.C06b*) retained strong enrichment of repressive H3K27me3 marks (Fig. S9d-f). Similarly, other floral meristem or floral organ identity genes, like *BnLFY* (Fig. S10a-c) and *BnAG* (Fig. S10d-f), which are not expressed in leaves, carried high levels of H3K27me3. Interestingly, two floral repressors were among the genes exhibiting differential histone mark enrichment between time points, although their overall patterns were similar between the genotypes. These were *BnTEM1* (Fig. S11a-c) and *BnSMZ* (Fig. S11d-f), both known as direct repressors of *FT* in Arabidopsis (Castillejo and Pelaz 2008; Mathieu et al. 2009). *BnTEM1.A09* displayed strong enrichment of H3K4me3 in week 2, which markedly decreased at later stages. A similar trend occurred for *BnTEM1.C05*. In contrast, *BnTEM1.A08* and *BnTEM1.C03* retained H3K4me3 enrichment. All four *BnTEM1* homologs displayed a modest increase in H3K27me3 at later stages. *BnSMZ.A09* carried H3K4me3 marks at week 2, followed by accumulation of repressive H3K27me3 marks at week 5 and week 6 in both genotypes. *BnSMZ.C08* showed a similar pattern. Overall, these results demonstrate that multiple genes controlling flowering time and floral organogenesis at the SAM, including both activators and repressors, are subject to epigenetic regulation in the leaves of the spring oilseed rape cultivar Westar.

## Discussion

In the allotetraploid crop *B. napus* different growth types with specific vernalization requirements have evolved, making it crucial to understand the molecular mechanisms behind this variation to adapt flowering time to different environmental conditions. A key regulator of vernalization is *FLC*, a well-characterized gene in Arabidopsis (Sheldon et al. 1999; Michaels and Amasino 1999) that has also been identified in *B. napus*. There are nine *FLC* homologs in *B. napus*, each with distinct expression patterns across different accessions and growth types (Song et al. 2020; Matar et al. 2021; Calderwood et al. 2021; Jones et al. 2024). The presence and expression levels of these homologs are linked to the differentiation between winter and spring types. Interestingly, *BnFLC* genes are present in winter types and spring types, with the latter lacking a vernalization requirement (Song et al. 2020; Yin et al. 2020). Understanding the specific role of each homolog will provide insights into the regulation of flowering time and vernalization in *B. napus*. This knowledge could ultimately enable fine-tuning of flowering time across growth types to optimize adaptation to various environmental conditions. To explore this, we generated CRISPR/Cas9 knockout mutants of all *BnFLC* genes in the spring cultivar Westar, revealing their impact on flowering time in spring types.

### Flowering time is affected by *BnFLC* homologs to varying degrees

Using an efficient transformation protocol (Ille and Melzer 2025), we generated mutants with various combinations of edited *BnFLC* genes. This approach allowed us to analyze not only the effect of a complete loss of all *BnFLC* genes but also the contribution of individual homologs. The mutant *flc-W6-1*, with knockouts in most *BnFLC* genes, flowered after 5 weeks, 1 week earlier than the wild type, while mutants with fewer mutated *BnFLC* genes flowered progressively later. These results suggest partial functional redundancy among the *BnFLC* genes, as mutants with fewer knockouts flowered significantly later than *flc-W6-1*.

In the mutant *flc-W6-1*, almost no *BnFLC* transcripts were detectable. This reduction may be explained by the activity of the nonsense-mediated mRNA decay (NMD) pathway. NMD is a conserved eukaryotic RNA surveillance mechanism that degrades aberrant transcripts and plays important roles in plant development (Rayson et al. 2012; Lykke-Andersen and Jensen 2015; Raxwal and Riha 2023; Panigrahi et al. 2026). The mutated *BnFLC* transcripts might trigger the NMD machinery, resulting in their degradation.

Interestingly, the *BnFLC.C02* homolog had a stronger effect on flowering time than other homologs, despite not having the highest expression level in leaves. This pronounced effect may reflect a specialized interaction in specific tissues that influence flowering time, which is not apparent from leaf expression data. Although *BnFLC.C02* has been described as cold responsive and variation in its expression has been reported among accessions differing in growth type or flowering time (Chen et al. 2018; Schiessl et al. 2019; Song et al. 2020; Jones et al. 2024), larger population studies have not consistently detected differential expression or a clear association with growth type. In these analyses, *BnFLC.A10* often showed a stronger effect and better discrimination between growth types (Wu et al. 2019; Schiessl et al. 2019; Calderwood et al. 2021). Notably, Song et al. (2020) found that the earlier flowering cultivar No2127, one of two spring cultivars including Westar, lacked *BnFLC.C02*. This aligns with our findings, that the loss of *BnFLC.C02* contributes most strongly to the early flowering phenotype of the mutant *flc-W6-1*. To some extent, *BnFLC.C09b* also appeared to influence flowering time, as *flc-W1-6* flowered significantly later than *flc-W6-1*, differing genotypically only for *BnFLC.C09b* (Fig. 2). Furthermore, *BnFLC.C09b* was not downregulated during flowering. Its upregulation during development has been reported across different growth types and a neofunctionalization of this gene has been proposed (Schiessl et al. 2019; Matar et al. 2021; Calderwood et al. 2021; Jones et al. 2024). However, despite this observed increase in transcript levels, it remains unclear whether this upregulation is reflected at the protein level and whether the protein functions as a floral repressor under the analyzed conditions. This may explain why the knockout of *BnFLC.C09b* did not result in a strong effect on flowering time.

Interpretation of individual *BnFLC* mutant phenotypes is complicated by the likely presence of unequal redundancy among *BnFLC* homologs. Following disruption of one homolog, remaining copies may partially compensate via altered expression levels as active compensation or will show a passive compensation without change in expression. Such mechanisms are thought to buffer perturbations in gene dosage (Iohannes and Jackson 2023). Consistent with this concept, a dosage-dependent effect of *BnFLC* genes on vernalization requirement has previously been proposed (Calderwood et al. 2021). Nevertheless, the distinct flowering phenotypes observed among individual *flc* mutants in the spring-type background suggest that *BnFLC* homologs contribute differently to flowering time regulation, despite evidence of partial compensation among remaining homologs.

In our greenhouse experiment, we observed differences between summer and winter conditions in terms of both absolute flowering time and the magnitude of change in mutants. This indicates that other factors like photoperiod, light intensity and temperature substantially affect flowering time, which was described for spring types (Salisbury and Green 1991; Rahman et al. 2018; Abelenda et al. 2023) and particularly in Westar (Yin et al. 2020; Ahmar et al. 2022). Therefore, the absolute effect of *BnFLC* gene knockouts under field conditions may vary considerably depending on location and growing conditions.

### Knockout of *BnFLC* genes alters the expression of multiple flowering time regulators in leaves

In line with the earlier flowering observed in the mutant, we noted an earlier upregulation of flowering time affecting genes in its leaves. Differential expression was not only detected for flowering time regulators known to be expressed in leaves such as *FT* (Corbesier et al. 2007), but also for genes primarily associated with the SAM, like *SOC1* or *SEP* genes (Ditta et al. 2004; Lee and Lee 2010). The earlier upregulation of *SOC1*,* FUL* and *FT* correlated with earlier flowering of the mutants (Fig. S3). While *SOC1* primarily affects flowering through the SAM, its upregulation in leaves, where its role remains unclear, has also been reported in Arabidopsis (Hepworth et al. 2002; Searle et al. 2006; Lee and Lee 2010). In oilseed rape, upregulation was described in both the SAM and leaves, but distinct homologs were activated depending on the tissue and genotype, suggesting subfunctionalization (Matar et al. 2021; Jones et al. 2024). Notably, the effect on transcript levels was most pronounced for *BnFT* genes, especially in the cauline leaves, and particularly for *BnFT.A02* and *BnFT.C06*, which were markedly higher in the mutant, likely due to the complete loss of all *BnFLC* genes. These *BnFT* genes are strongly linked to flowering time and growth type diversification in oilseed rape (Tudor et al. 2020; Wan et al. 2025; Yin et al. 2025). The heightened expression of *BnFT* genes in the mutant *flc-W6-1* suggests that BnFLC normally represses *BnFT* expression in Westar. However, the varied responses of *BnFT* homologs indicate that their regulation may differ, pointing to a more complex mechanism than simple BnFLC-mediated repression. In addition, the earlier expression of *BnFUL* genes in mutant leaves likely results from the elevated *BnFT* expression, as FT-dependent accumulation of *FUL* transcripts in leaves has been described in Arabidopsis (Teper-Bamnolker and Samach 2005).

Earlier flowering in the mutant may also be influenced by altered GA levels, as two *BnGA20OX3* genes were upregulated in week 1 (Fig. S5). These genes are involved in the biosynthesis of bioactive GAs, and knockouts of *GA20OX* genes in Arabidopsis lead to dwarfism and delayed flowering (Plackett et al. 2012). Additionally, BnFLC proteins may directly affect GA signaling pathways, as genes in the GA pathway have been identified as targets of FLC in Arabidopsis (Deng et al. 2011). The BR hormone signaling might be also affected, as some *BnHBI1* and *BnBEE2* homologs are upregulated. *HBI1* and *BEE2* are described as close homologs in *Arabidopsis* and are basic helix-loop-helix transcription factors. They are working as activators of BR signalling/biosynthesis. They are described as GA- and BR- responsive and important for cell elongation and thereby hypocotyl elongation (Friedrichsen et al. 2002; Bai et al. 2012; Malinovsky et al. 2014). The early and high upregulation of these genes in the mutant might be a result of a different hormonal status of the plant, which lead to a faster development.

### Expression of flowering time regulators is strongly affected by epigenetic modifications

In addition to flowering time and floral transition genes, those controlling floral meristem and floral organ identity, such as *BnSEP1*, *BnSEP2* and *BnSEP3*, were also differentially expressed. *BnSEP1* and *BnSEP2* were upregulated more than a week earlier in the mutant (Fig. S2), suggesting that this differential expression may not only reflect accelerated development, but also a direct effect of BnFLC on *BnSEP1* and *BnSEP2* expression. In Arabidopsis, *SEP3* has been identified as a direct target of FLC (Deng et al. 2011) and shows FT-dependent upregulation in leaves (Teper-Bamnolker and Samach 2005), while *SEP1* and *SEP2* are not expressed in leaves (Ma et al. 1991). The observed expression differences were accompanied by changes in histone marks, including the removal of H3K27me3 and the deposition of H3K4me3. This indicates that *BnSEP* genes are epigenetically regulated during development, with the loss of *BnFLC* functions altering the timing of this regulation. In Arabidopsis, loss of H3K27me3 at *SEP* loci leads to misexpression and a leaf phenotype (Kotake et al. 2003; Mateo-Bonmatí et al. 2018). In oilseed rape, activation of *BnSEP* genes in leaves also occurs in the wild type, suggesting that these genes may have additional functions in leaves.

In contrast, the *BnFLC* genes expressed in Westar do not appear to be epigenetically regulated during development. We did not detect H3K4me3 marks at any of the *BnFLC* loci and clear H3K27me3 marks were only observed for *BnFLC.A03b* (Fig. S7) which is a candidate for growth type differentiation (Schiessl et al. 2019). No large structural variants, such as those reported for *BnFLC.A02* and *BnFLC.A10* (Song et al. 2020; Yin et al. 2020), were identified, implying that epigenetic silencing may be responsible for the undetectable expression in Westar. In contrast, activating histone marks have been detected in winter types for *BnFLC.C02* and *BnFLC.A03b* before winter (Lu et al. 2022), and their absence in Westar could contribute to differences in the regulation of *BnFLC* genes and their expression levels. Similarly, in Arabidopsis, the rapid-cycling accession Col-0 exhibits fewer activating histone marks (H3K4me3/H3K36me3) compared to winter annuals with a functional *FRI* gene, while the *FLC* locus in Col-0 showed increased H3K27me3 marks (Ding et al. 2013). Therefore, it would be valuable to investigate whether general differences in the epigenetic regulation of *BnFLC* genes across accessions exist in both leaves and meristems, and how they may influence growth type differentiation.

We observed that several flowering regulators, including both activators and repressors, are expressed in leaves and exhibit dynamic epigenetic regulation. These include *BnSEP1*, *BnSEP2*, *BnSOC1*, *BnFUL* and *BnTEM1*. In Arabidopsis, epigenetic regulation has been described for several of these genes, although not always across all tissues and developmental stages. For instance, in the SAM of Arabidopsis, *SOC1* acquires H3K4me3 marks during floral transition while H3K27me3 levels remain unchanged (You et al. 2017). Our results suggest that *BnSOC1* expression in Westar is similarly influenced by active marks in the leaves, although repressive marks are lost at later developmental stages (Fig. S8d-f). In contrast, floral regulators primarily active in meristems, such as *AG* and *LFY* (Liljegren et al. 1999; Lohmann et al. 2001), were not expressed in oilseed rape leaves and showed strong repressive H3K27me3 histone marks. This indicates that epigenetic regulation silences their expression in leaves. In Arabidopsis, ectopic expression of *AG* in leaves occurs in Polycomb group protein mutants, due to the loss of H3K27me3 marks, resulting in a curled leaf phenotype (Schubert et al. 2006; Adrian et al. 2009). We also observed tissue-specific epigenetic regulation in *BnSPL15* and *BnAP1* (Fig. S9). For *BnSPL15.C04*, activating histone marks were present throughout development, and its transcript levels steadily increased in the leaves of wild-type plants up to flowering. Notably, this homolog did not show increased expression in the SAM during vernalization in winter type oilseed rape (Matar et al. 2021). In Arabidopsis, *SPL15* controls flowering in short days and influences leaf initiation rates as well as leaf cell size and number (Schwarz et al. 2008; Usami et al. 2009). The leaf-specific upregulation of *BnSPL15.C04* implies it may have a regulatory role in leaves. In contrast, all other *BnSPL15* homologs exhibited lower expression and were associated with repressive H3K27me3 marks, indicating homolog-specific activation and silencing in leaves. Similarly, *BnAP1*.*C06b* carried strong repressive marks throughout development and remained unexpressed in leaves, whereas two other homologs displayed strong expression in leaves (Fig. S4c, d) and lacked repressive marks.

Finally, our results demonstrate that *BnFLC* genes are developmentally regulated in spring oilseed rape, unlike in winter types, where cold exposure primarily affects *BnFLC* expression. Analyzing *BnFLC* genes in spring types does not fully resolve the extent to which individual homologs contribute to growth type differentiation. In addition to the well-known candidate genes *BnFLC.A02* and *BnFLC.A10*, which are not expressed in Westar, *BnFLC.A03b* emerges as a strong candidate due to its specific epigenetic silencing. Our findings suggest that epigenetic regulation in oilseed rape might play an important role in growth type differentiation. Beyond this, the functional relevance of flowering regulators expressed in leaves, aside from *BnFLC*, remains an open question. These genes may not simply be aberrantly expressed but could serve broader or redundant roles in coordination with other regulators. Moreover, their temporal and spatial expression might be influenced not only by specific transcription factors but also by chromatin organization and histone modifications, as proper epigenetic regulation is crucial for plant development (Zhang et al. 2009; Pikaard and Mittelsten Scheid 2014; Nakamura et al. 2026). Thus, epigenetic regulation may play a key role in the subfunctionalization of homologs in *B. napus*, as evidenced by the tissue-specific regulation observed in our study.

## Supplementary Information

Below is the link to the electronic supplementary material.

Supplementary Material 1

## Data Availability

Raw RNA-seq and CUT&Tag sequencing reads have been deposited in the NCBI Sequence Read Archive under BioProject ID PRJNA1393645.
