# Supplementary material for: FLC genes control flowering time to varying degrees in a Brassica napus spring cultivar
Source: Plant Mol Biol. 2026 Jul 29;116(4):79. doi: 10.1007/s11103-026-01741-7 (PMC13421269; doi:10.1007/s11103-026-01741-7)
Supplement: Supplementary file 1 — Supplementary Material 1 [file 11103_2026_1741_MOESM1_ESM.docx]

***FLC* genes control flowering time to varying degrees in a *Brassica napus* spring cultivar**

Sarah Duveneck, Kea Ille and Siegbert Melzer

Plant Developmental Biology and Physiology, Kiel University, Am Botanischen Garten 5, 24118 Kiel, Germany

Siegbert Melzer ([smelzer@bot.uni-kiel.de](mailto:smelzer@bot.uni-kiel.de))

Sarah Duveneck ([sduveneck@bot.uni-kiel.de](mailto:sduveneck@bot.uni-kiel.de))

Kea Ille ([kille@bot.uni-kiel.de](mailto:kille@bot.uni-kiel.de))

ORCID iD: Sarah Duveneck (0009-0008-2540-1819)

ORCID iD: Kea Ille (0009-0005-2758-2623)

ORCID iD: Siegbert Melzer (0000-0003-4008-4782)

**Fig. S1** Verification of the presence of two *BnFLC.A03a* genes in Westar. (a) Gene structure of the *BnFLC.A03a* genes from exon1 to exon 2, showing the position of the InDel and the primers used. Primers spanning the InDel were designed to distinguish the two genes. Expected PCR fragment sizes are 345 bp for *BnFLC.A03a.1* and 319 bp for *BnFLC.A03a.2*. (b) PCR was performed on Westar wild type and the mutant *flc-W4-1*, which carries no mutations in the *BnFLC.A03a* genes. Both showed the expected fragment sizes. The GeneRuler 100 bp was used.

**
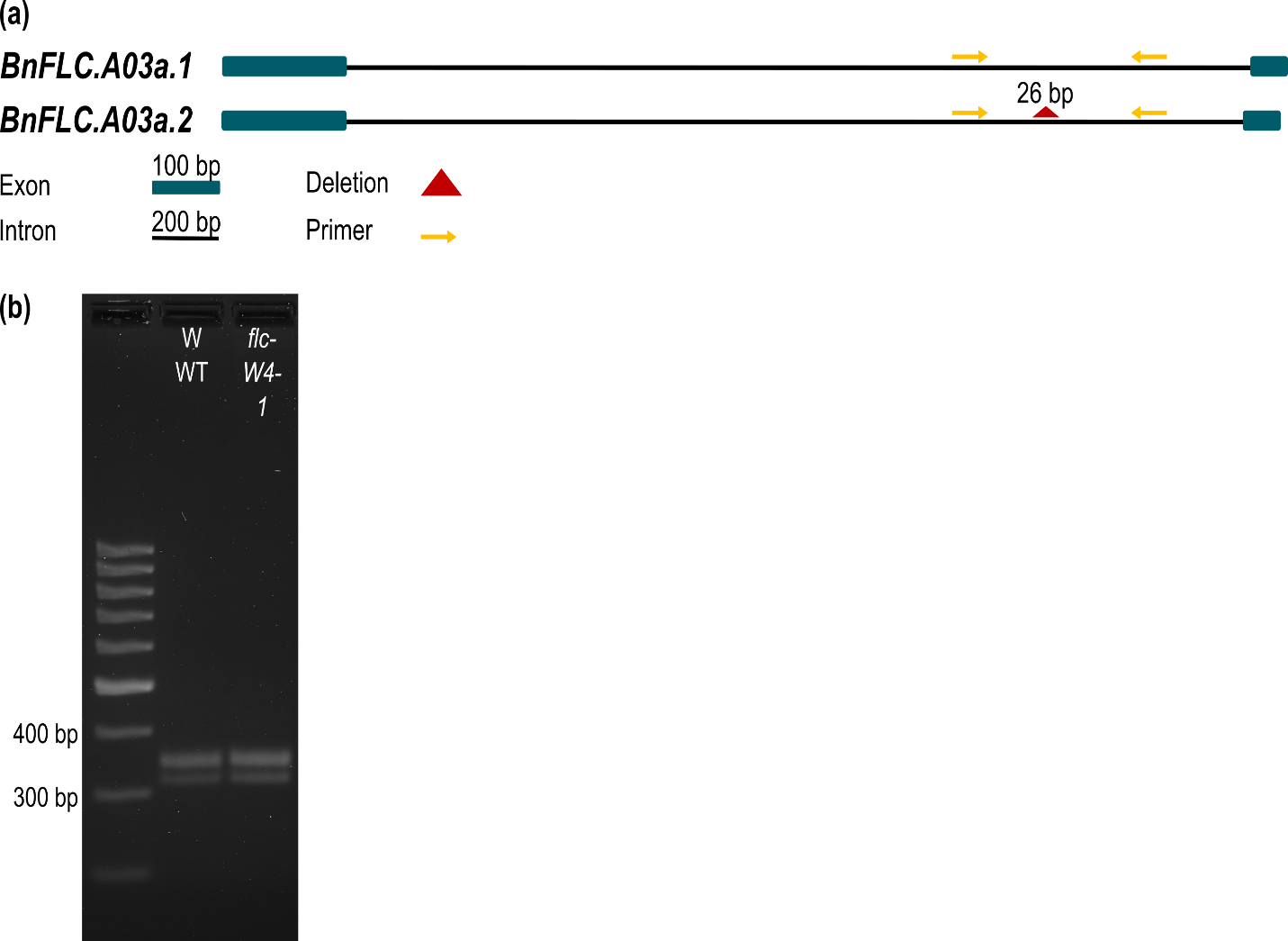
**

**
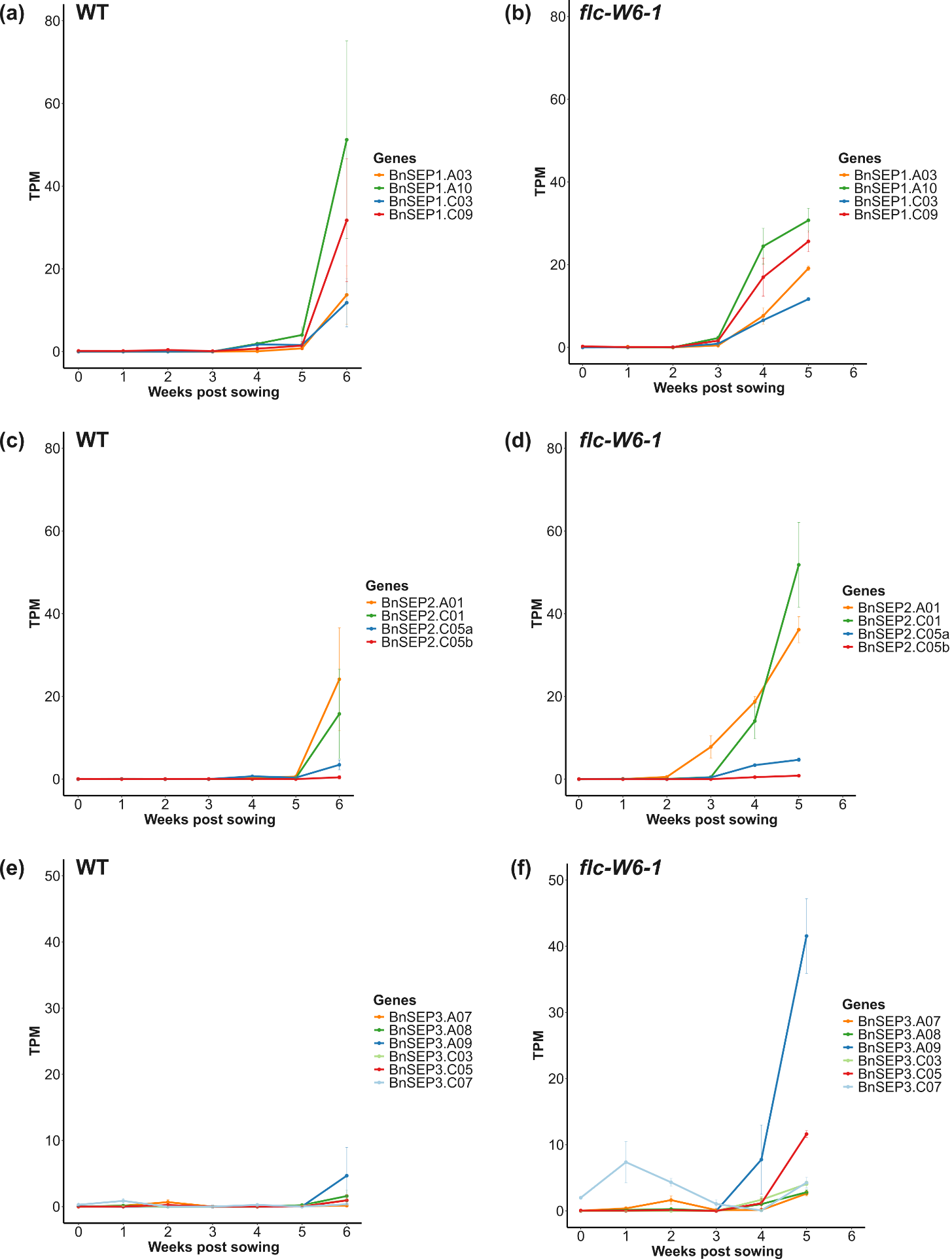
Fig. S2** Expression levels of *BnSEP1* (a,b), *BnSEP2* (c,d) and *BnSEP3* (e,f) genes in Westar wild type and in the mutant *flc-W6-1* in the leaves. Expression values are shown as TPM ± SD.

**
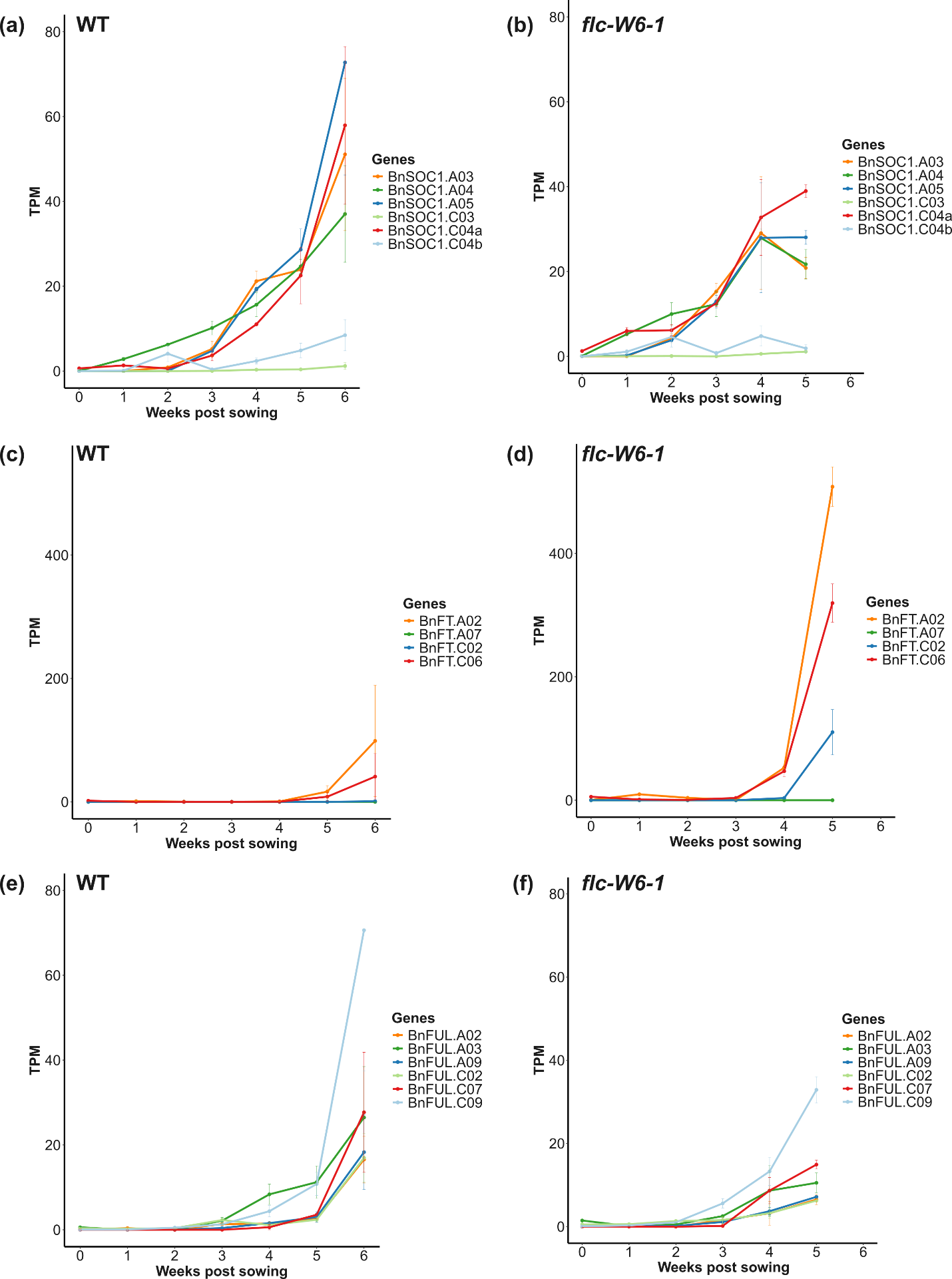
Fig. S3** Expression levels of *BnSOC1* (a,b), *BnFT* (c,d) and *BnFUL* (e,f) genes in Westar wild type and in the mutant *flc-W6-1* in the leaves. Expression values are shown as TPM ± SD.

**
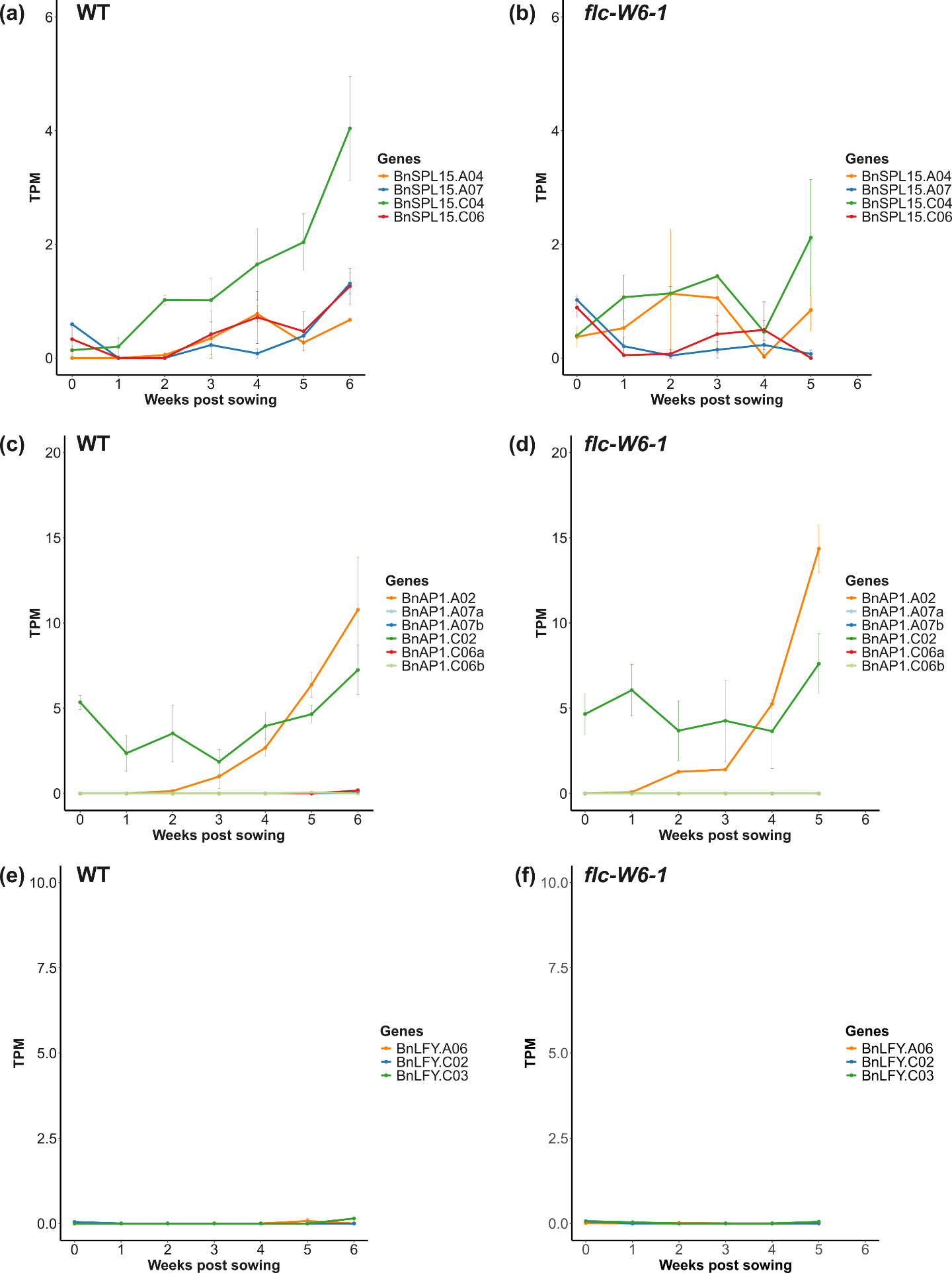
Fig. S4** Expression levels of *BnSPL15* (a,b), *BnAP1* (c,d) and *BnLFY* (e,f) genes in Westar wild type and in the mutant *flc-W6-1* in the leaves. Expression values are shown as TPM ± SD.

**
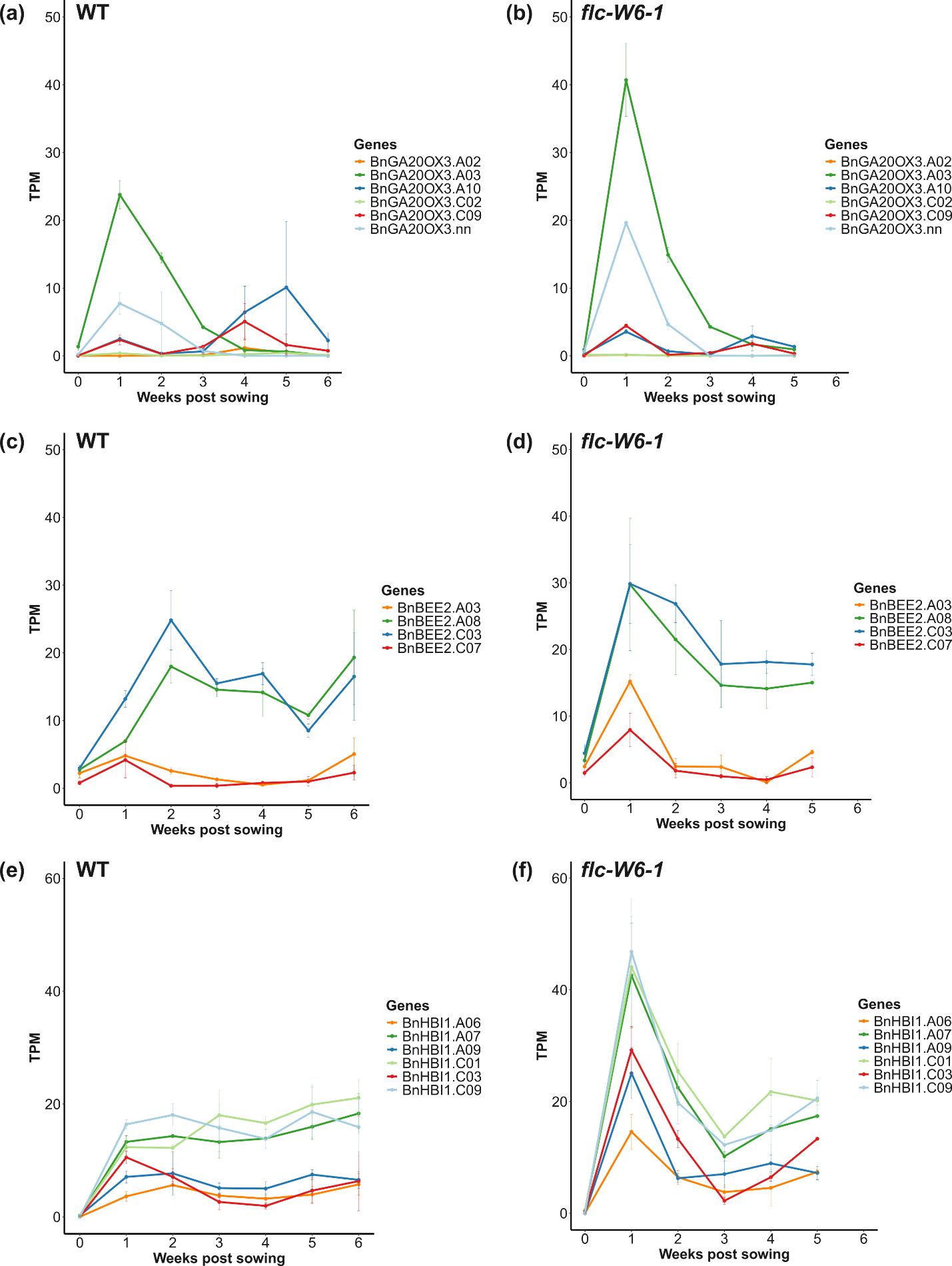
Fig. S5** Expression levels of *BnGA20OX3* (a,b), *BnBEE2* (c,d) and *BnHBI1*(e,f) genes in Westar wild type and in the mutant *flc-W6-1* in the leaves. Expression values are shown as TPM ± SD.

**Fig. S6** CUT&Tag analysis of histone marks (a) Representative heatmaps showing enrichment of H3K27me3 and H3K4me3 relative to annotated transcription start site (TSS) and transcription end sites (TES). Signal intensities are plotted from 3 kb upstream of the TSS to 3 kb downstream of the TES. The heatmaps display individual genes as rows, sorted by signal strength. Color scale represents normalized signal intensity, ranging from low (red) to high (blue).
(b) Heat map showing the correlation of histone mark profiles across all samples for both H3K27me3 and H3K4me3 marks.

**
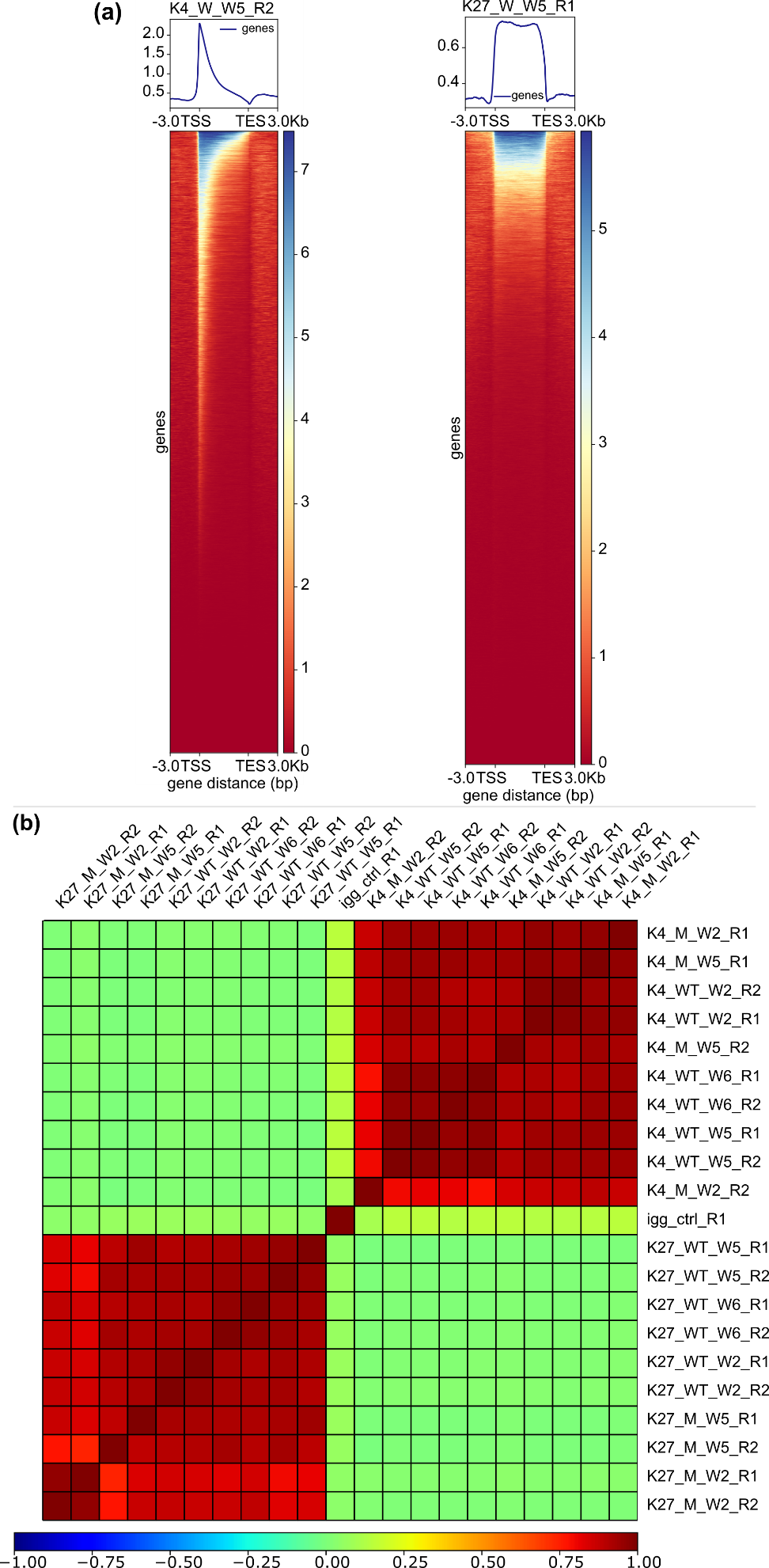
**

**Fig. S7** Histone marks of selected *BnFLC* genes in Westar wild type and the mutant *flc-W6-1*.

H3K4me3 and H3K27me3 enrichment across the *BnFLC* genes in leaves by showing *BnFLC.A03b* (a,b), *BnFLC.C09b* (c,d), *BnFLC.C02* (e,f), *BnFLC.A03a.1* (g,h), *BnFLC.A03a.2* (i,j), and *BnFLC.C09a* (k,l). For histone marks two replicates per time point are displayed. IgG is shown as negative control.


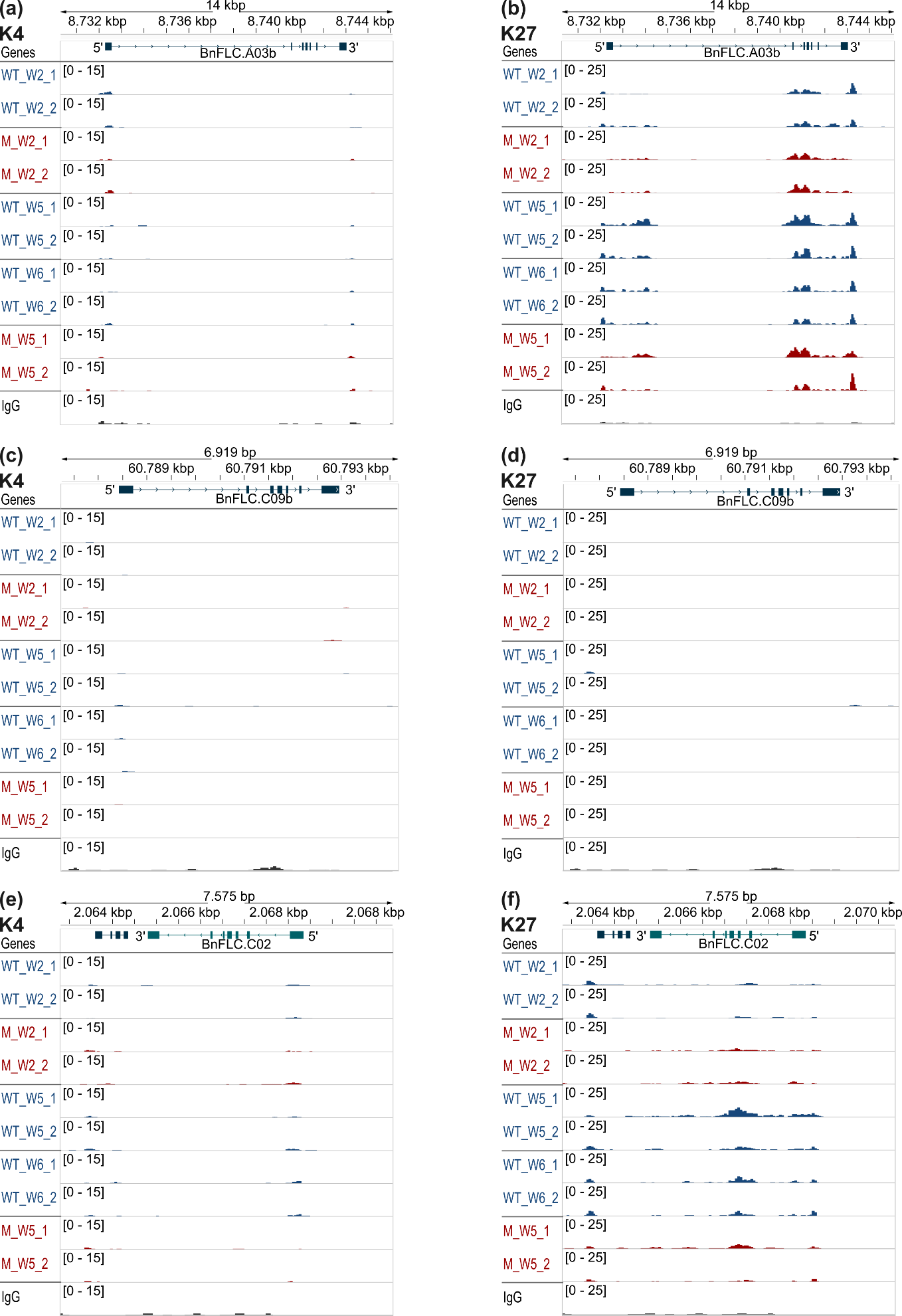


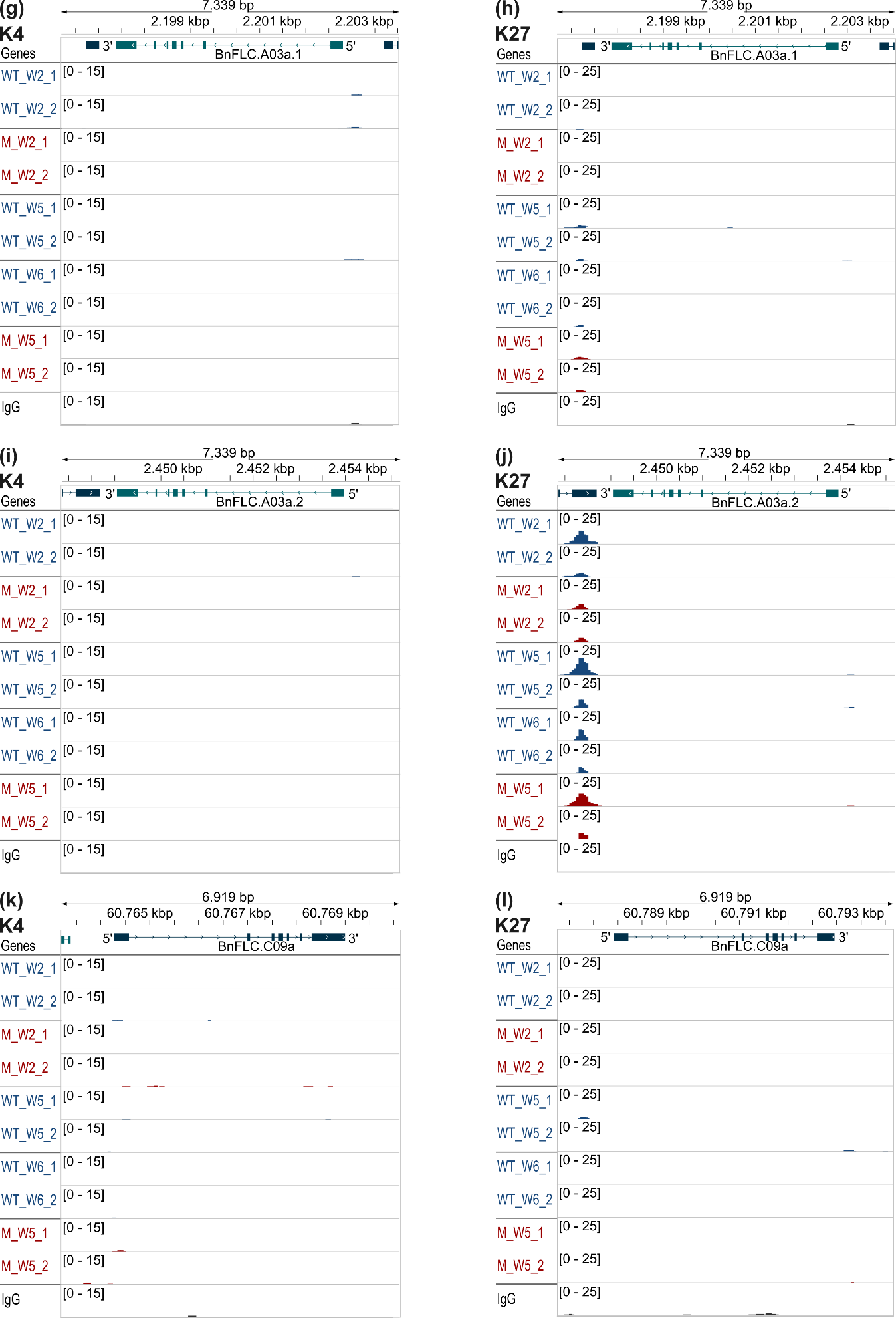


**Fig. S8** Expression profiles and histone marks of *BnFUL.C07* and *BnSOC1.A03* in Westar wild type and the mutant *flc-W6-1*.

(a–c) *BnFUL.C07*: expression profile (a) and histone marks H3K4me3 and H3K27me3 (b,c) in leaves.
(d–f) *BnSOC1.A03*: expression profile (d) and histone marks H3K4me3 and H3K27me3 (e,f) in leaves.

Expression values are shown as TPM ± SD. For histone marks two replicates per time point are displayed. IgG is shown as negative control.


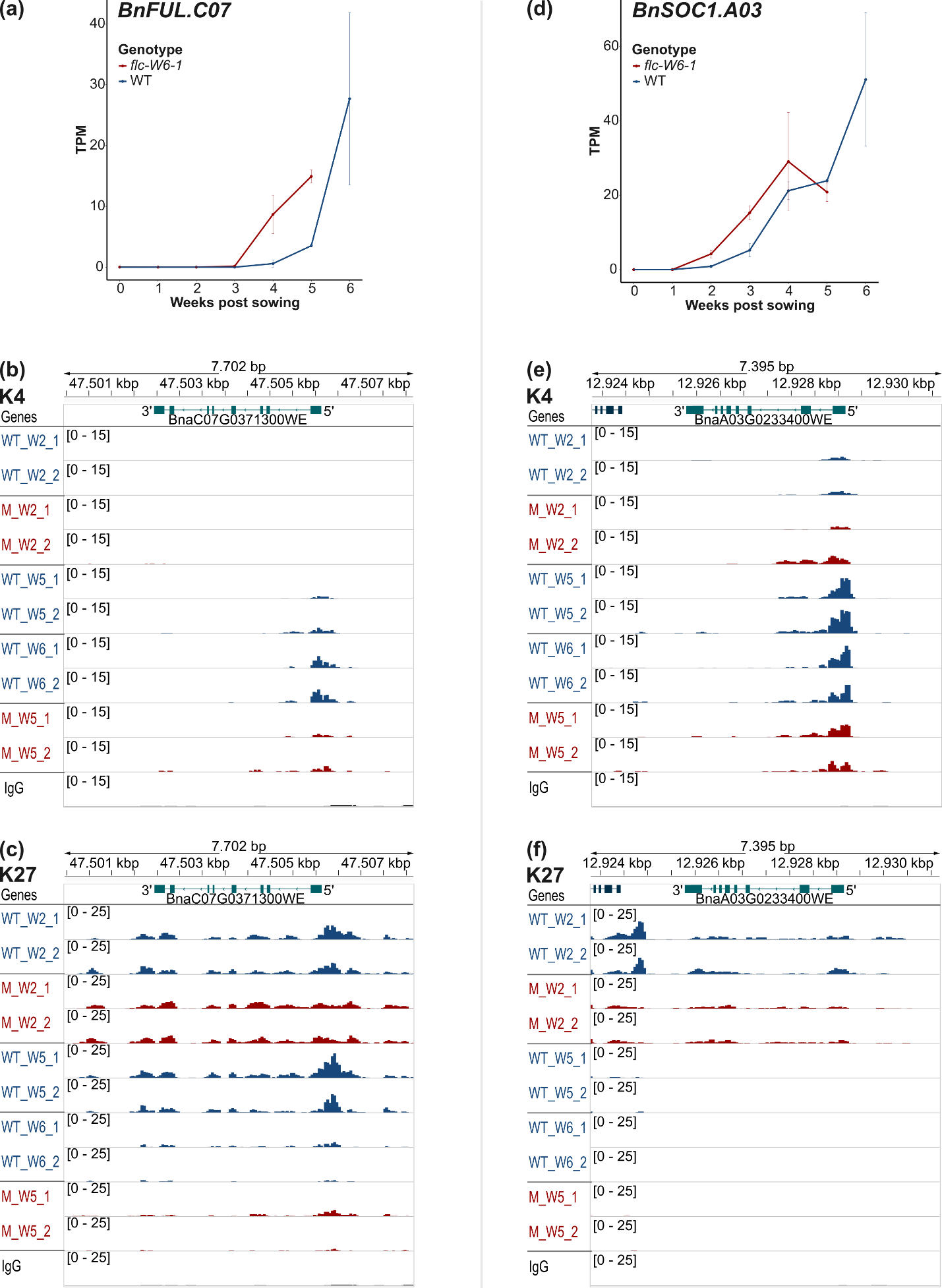


**Fig. S9** Expression profiles and histone marks of *BnSPL15.C04* and *BnAP1.C06b* in Westar wild type and the mutant *flc-W6-1*.

(a–c) *BnSPL15.C04*: expression profile (a) and histone marks H3K4me3 and H3K27me3 (b, c) in leaves.
(d–f) *BnAP1.C06b:* expression profile (d) and histone marks H3K4me3 and H3K27me3 (e, f) in leaves.

Expression values are shown as TPM ± SD. For histone marks two replicates per time point are displayed. IgG is shown as negative control.


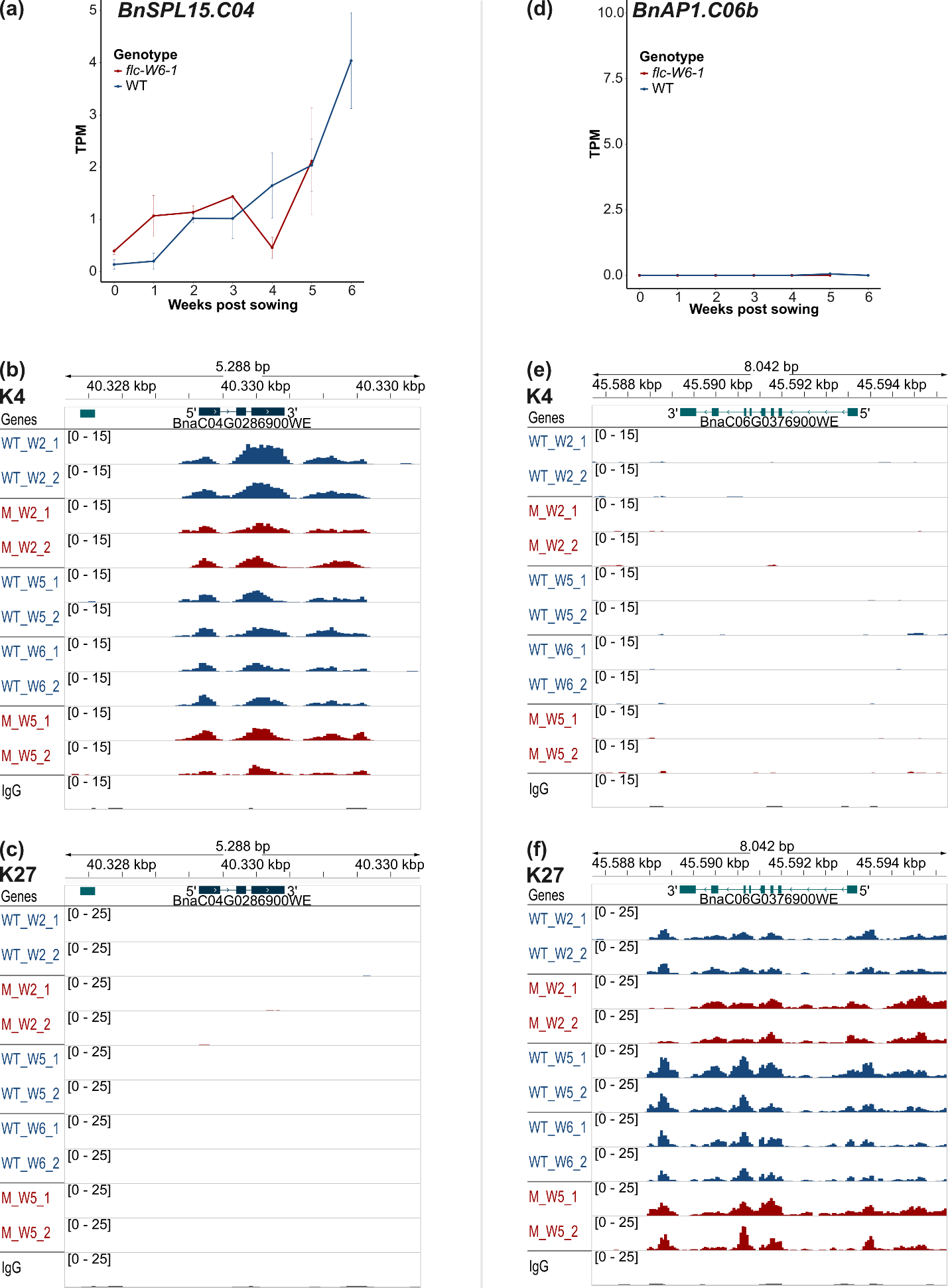


**Fig. S10** Expression profiles and histone marks of *BnLFY.A06* and *BnAG.C01* in Westar wild type and the mutant *flc-W6-1*.

(a–c) *BnLFY.A06*: expression profile (a) and histone marks H3K4me3 and H3K27me3 (b,c) in leaves.
(d–f) *BnAG.C01:* expression profile (d) and histone marks H3K4me3 and H3K27me3 (e,f) in leaves.

Expression values are shown as TPM ± SD. For histone marks two replicates per time point are displayed. IgG is shown as negative control.

**
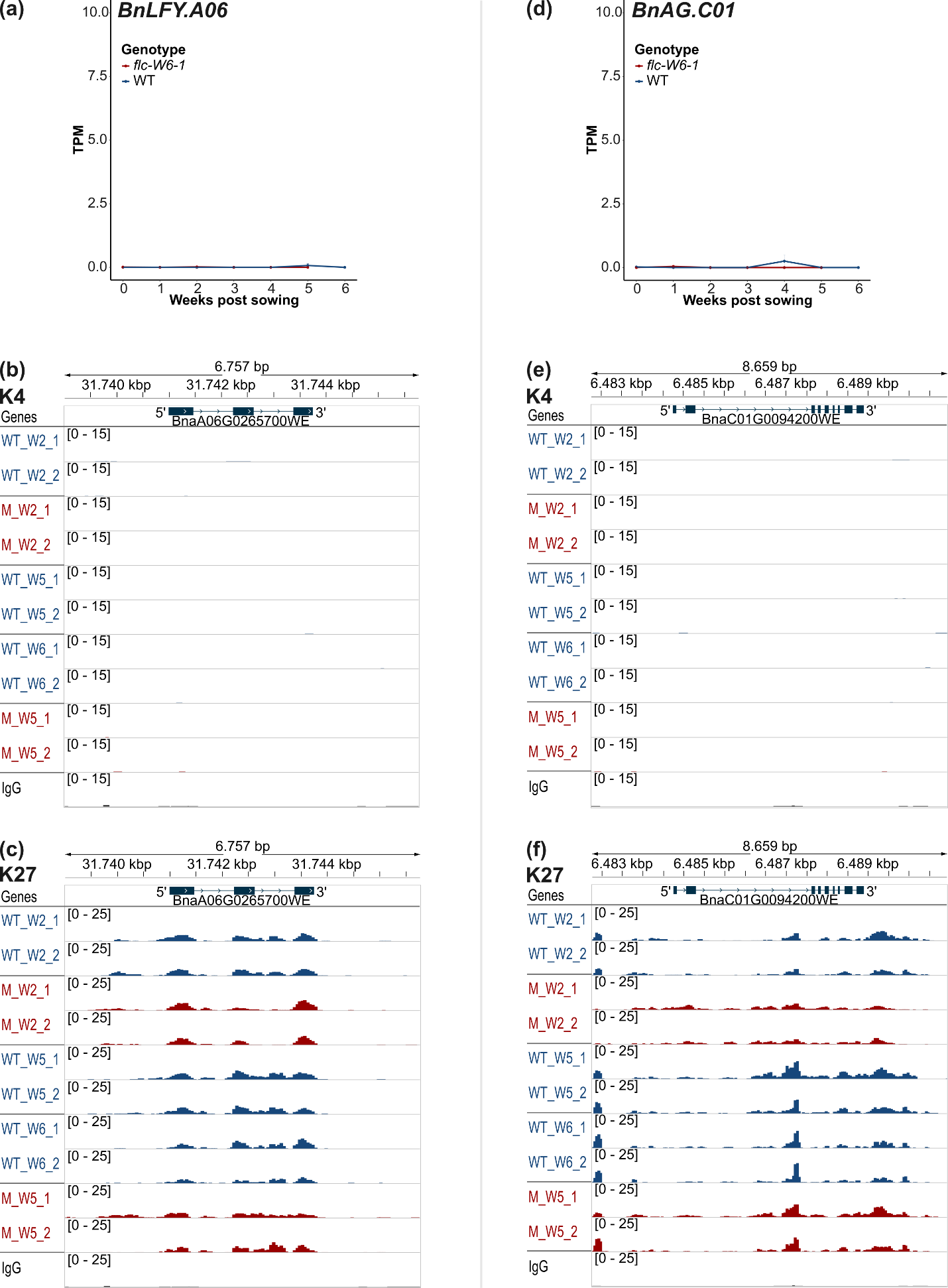
**

**Fig. S11** Expression profiles and histone marks of *BnTEM1.A09* and *BnSMZ.A09* in Westar wild type and the mutant *flc-W6-1*.

(a–c) *BnTEM1.A09*: expression profile (a) and histone marks H3K4me3 and H3K27me3 (b,c) in leaves.
(d–f) *BnSMZ.A09:* expression profile (d) and histone marks H3K4me3 and H3K27me3 (e,f) in leaves.

Expression values are shown as TPM ± SD. For histone marks two replicates per time point are displayed. IgG is shown as negative control.

**
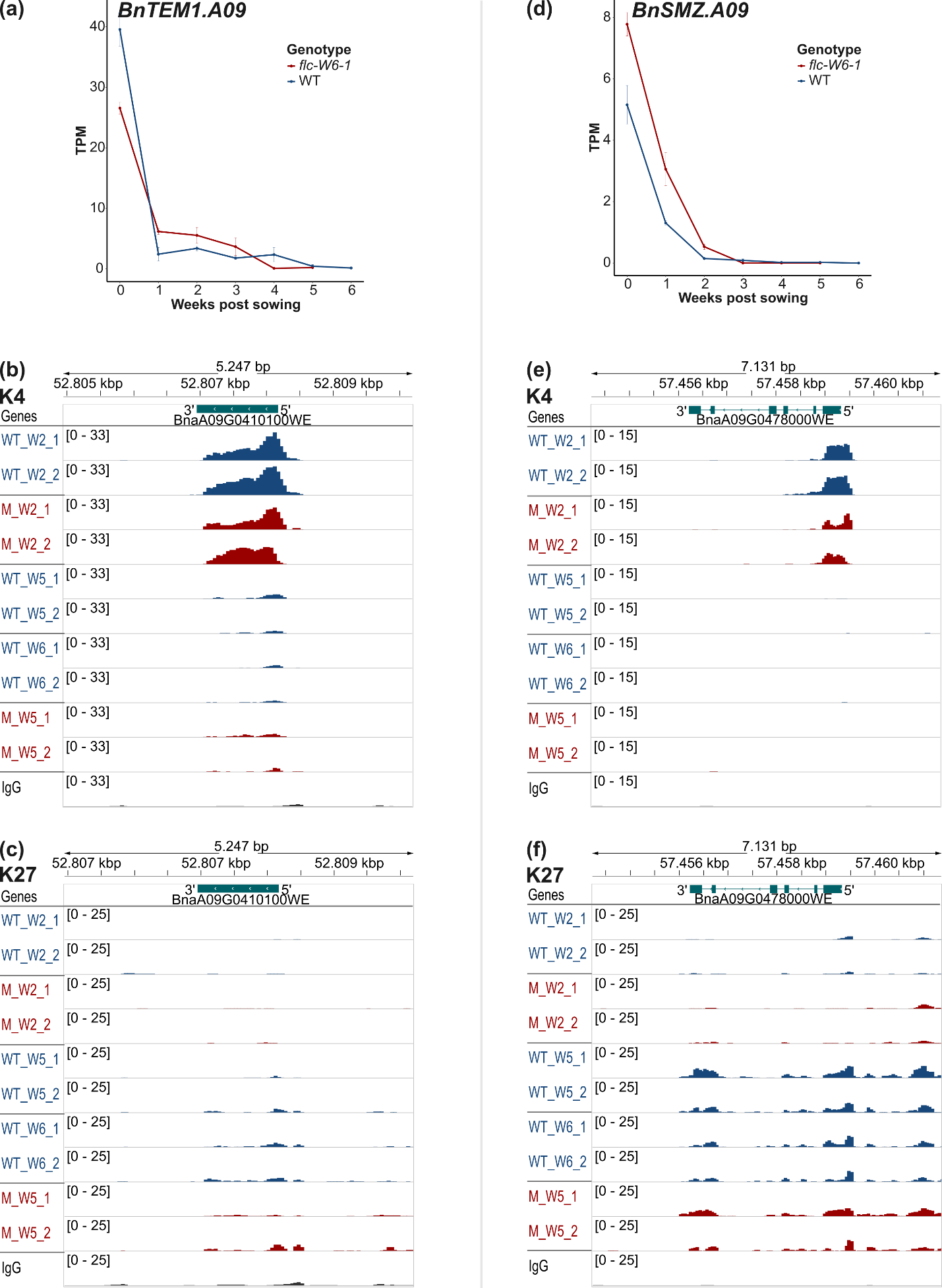
**

**Table S1** Gene identities (IDs) of BnFLC genes in Westar according to the reference genome (Song et al., 2020). Gene structures were manually verified and updated in the corresponding annotation file (Data S1) for subsequent transcriptomic and epigenetic analyses.

| **Gene IDs** | **Transcript**  **identifier** | **Synonym**  **(annotation file)** |
| --- | --- | --- |
| *BnFLC.A02* | A02T0035300 | FLC_A02 |
| *BnFLC.C02* | C02T0036300 | FLC_C02 |
| *BnFLC.A03a.1* | A03T0045800 | FLC_A03.1 |
| *BnFLC.A03a.2* | A03T0051700 | FLC_A03.2 |
| *BnFLC.A03b* | A03T0169800 | FLC_A03b |
| *BnFLC.C03b* | C03T0107800 | FLC_C03b |
| *BnFLC.C09a* | C09T0529200 | FLC_C09 |
| *BnFLC.C09b* | C09T0529500 | FLC_C09b |
| *BnFLC.A10* | A10T0297000 | FLC_A10 |

**Table S2** CRISPR/Cas9 target sites designed for *BnFLC* genes in Westar.

| **Target sites** | **Target genes** | **Sequence Target Site** | **PAM** | **Oligo for sgRNA construction** |
| --- | --- | --- | --- | --- |
| **TS1** | BnFLC.A02 | GTCAAGATCCTTGATCGATA | TGG | tgcaGTCAAGATCCTTGATCGATA |
|  | BnFLC.C02 | GTCAAGATCGTTGATCGATA | TGG | aaacTATCGATCAAGGATCTTGAC |
|  | BnFLC.C09a | GTCAAGATCCTTGATCGATA | TGG |  |
|  | BnFLC.C09b | GTCAAGATCCTTGATCGATA | CGG |  |
|  | BnFLC.A10 | GTCAAGATCCTTGATCGATA | TGG |  |
|  |  |  |  |  |
| **TS2** | BnFLC.A02 | TCATGGTGTGAACCATACTT | CGG | tgcaTCATGGTGTGAACCATACTT |
|  | BnFLC.C02 | TCATGGTGTGAACCATACTT | CGG | aaacAAGTATGGTTCACACCATGA |
|  |  |  |  |  |
| **TS3** | BnFLC.A03a.1 | GACACCCTCCAGCTGAACGA | GGG | tgcaGACACCCTCCAGCTGAACGA |
|  | BnFLC.A03a.2 | GACACCCTCCAGCTGAACGA | GGG | aaacTCGTTCAGCTGGAGGGTGTC |
|  |  |  |  |  |
| **TS4** | BnFLC.A03a.1 | AATCTCCGACATCAATCTTC | CGG | tgcaAATCTCCGACATCAATCTTC |
|  | BnFLC.A03a.2 | AATCTCCGACATCAATCTTC | CGG | aaacGAAGATTGATGTCGGAGATT |
|  | BnFLC.C09a | AATCTCCGACATCAATCTTC | CGG |  |
|  | BnFLC.C09b | AATCTCCGACATCAATCTTC | TGG |  |
|  | BnFLC.A10 | AATCTCCGACATCAATCTTC | CGG |  |
|  |  |  |  |  |
| **TS5** | BnFLC.A03b | GGTGATCTTCTAGCTCAACG | AGG | tgcaGGTGATCTTCTAGCTCAACG |
|  | BnFLC.C03b | GGTGATCTTCTAGCTCAACG | AGG | aaacCGTTGAGCTAGAAGATCACC |
|  |  |  |  |  |
| **TS6** | BnFLC.A03b | TCCGAGCTCTAGTTACAGAG | AGG | tgcaTCCGAGCTCTAGTTACAGAG |
|  | BnFLC.C03b | TCCGAGCTCTAGTTACAGAG | AGG | aaacCTCTGTAACTAGAGCTCGGA |


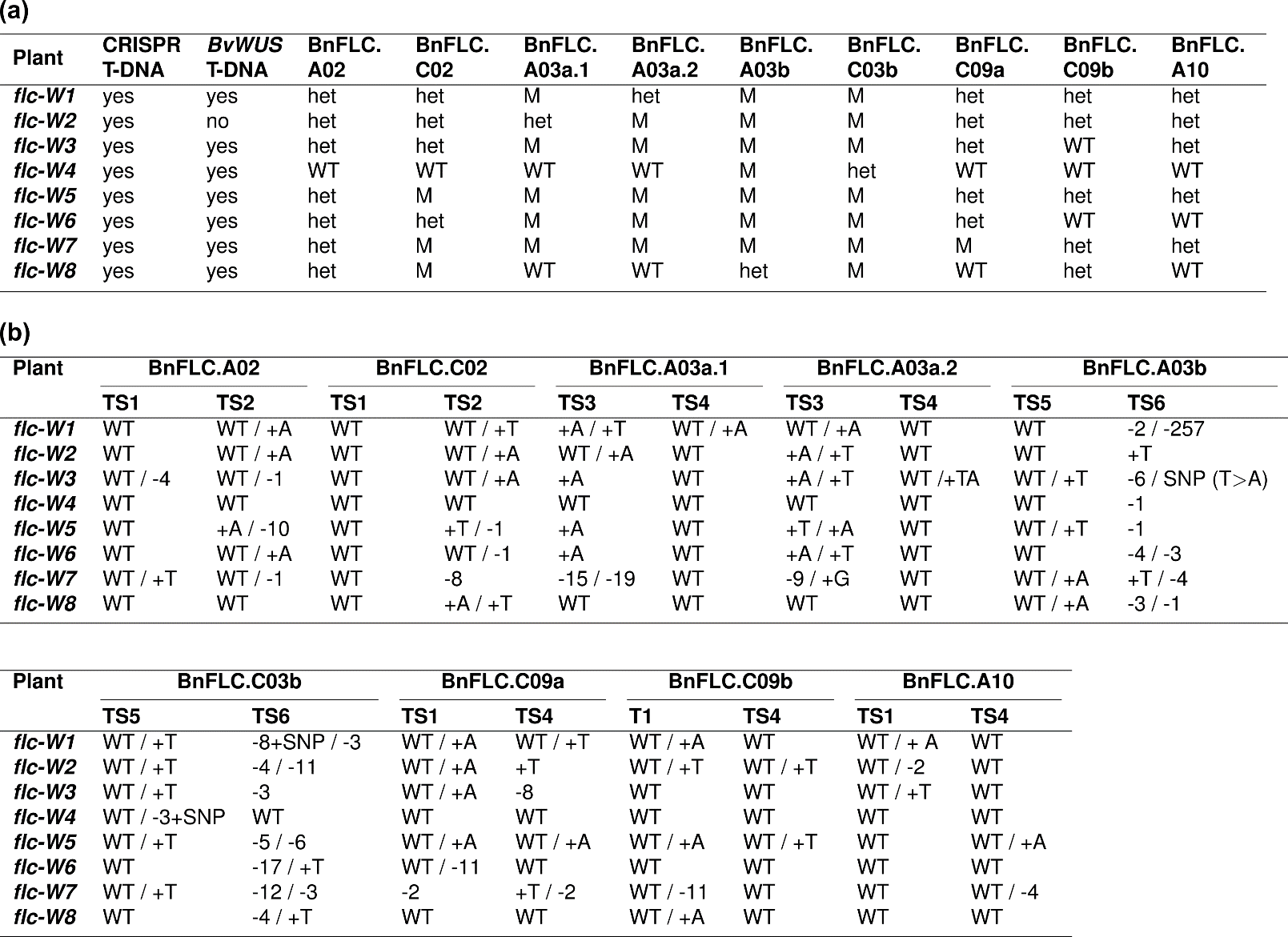
**Table S3** Summarized (a) and detailed (b) genotyping results of primary transformants. T-DNA/transgene integration was assessed by PCR. Mutations at each target site were identified by amplicon sequencing and Sanger sequencing. **“M” indicates a homozygous or biallelic mutation,** **“het” indicates a heterozygous genotype.**

**Table S4** Genotypes of mutants analyzed in this study. The days to flowering (DTF) were recorded in the greenhouse during summer.


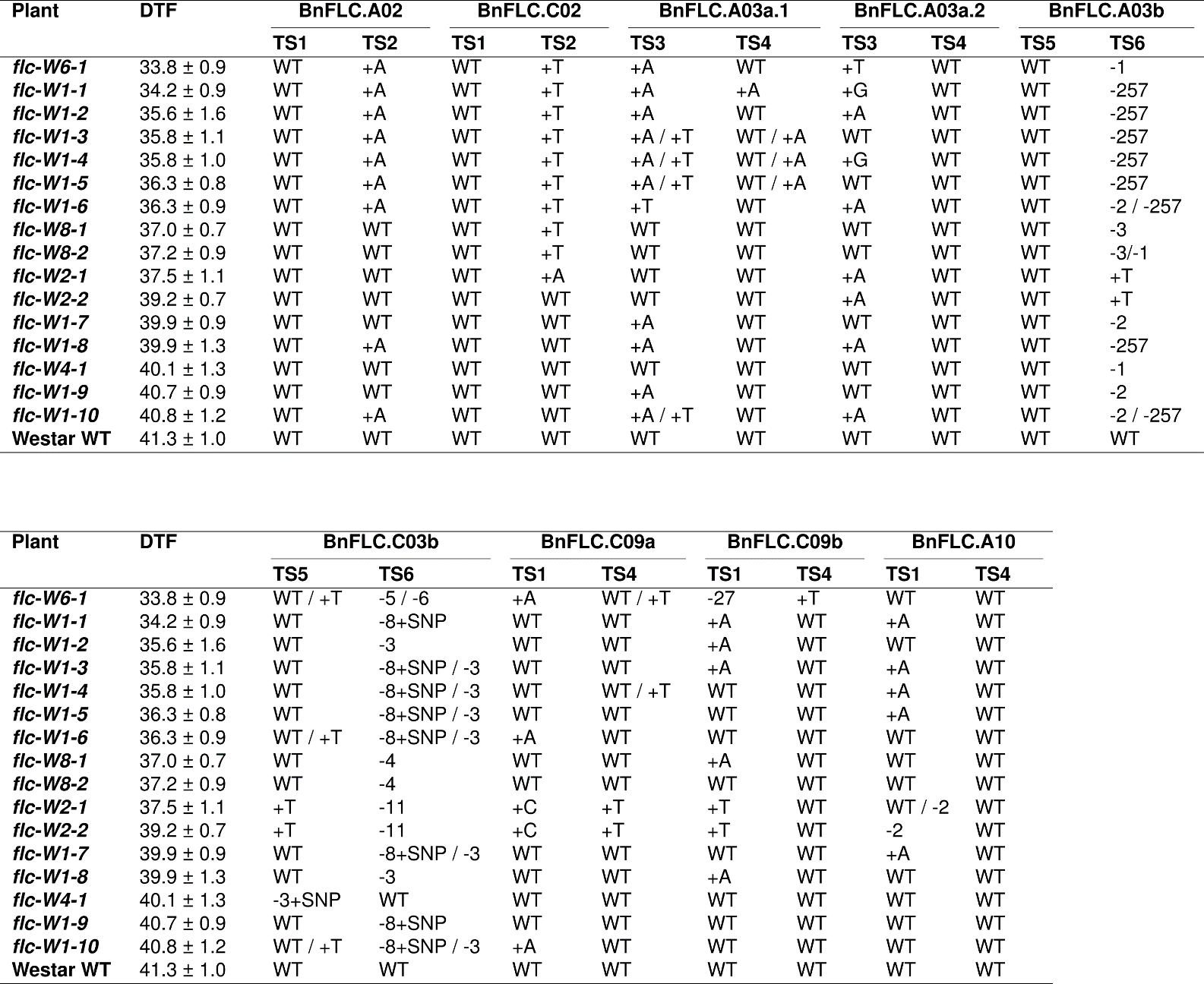


**Table S5** Flowering time of the mutant *flc-W6-1* and Westar wild type under different greenhouse and climate chamber conditions. The mutant consistently flowered earlier, with the difference varying between conditions.

| **Plant** | **Condition** | **DTF ± SD** |
| --- | --- | --- |
| *flc-W6-1* | greenhouse summer | 33.8 ± 0.9 |
| Westar WT | greenhouse summer | 41.3 ± 1.0 |
| *flc-W6-1* | greenhouse winter | 47.8 ± 1.6 |
| Westar WT | greenhouse winter | 61.8 ± 2.7 |
| *flc-W6-1* | climate chamber | 31.4 ± 1.5 |
| Westar WT | climate chamber | 42.8 ± 1.6 |

**Table S6** Gene names and IDs in Westar and Arabidopsis for genes analyzed in transcriptomic and epigenetic analyses.

| **Gene name Westar** | **Transcript ID**  **Westar** | | | **Gene name Arabidopsis** | | **Gene ID Arabidopsis** |
| --- | --- | --- | --- | --- | --- | --- |
|  |  | | |  | |  |
| BnAG.A01 | BnaA01T0019400WE | | | AG | | AT4G18960 |
| BnAG.A03 | BnaA03T0458200WE | | |  |  |  |
| BnAG.C01 | BnaC01T0094200WE | | |  |  |  |
| BnAG.C03 | BnaC03T0614700WE | | |  |  |  |
| BnAG.C06 | BnaC06T0178700WE | | |  |  |  |
|  |  | | |  | |  |
| BnAP1.A02 | BnaA02T0178100WE | | | AP1 | | AT1G69120 |
| BnAP1.A07a | BnaA07T0267300WE | | |  |  |  |
| BnAP1.A07b | BnaA07T0300600WE | | |  |  |  |
| BnAP1.C02 | BnaC02T0224300WE | | |  |  |  |
| BnAP1.C06a | BnaC06T0331200WE | | |  |  |  |
| BnAP1.C06b | BnaC06T0376900WE | | |  |  |  |
|  |  | | |  | |  |
| BnBEE2.A03 | BnaA03T0561800WE | | | BEE2 | | AT4G36540 |
| BnBEE2.A08 | BnaA08T0164700WE | | |  |  |  |
| BnBEE2.C03 | BnaC03T0602400WE | | |  |  |  |
| BnBEE2.C07 | BnaC07T0473500WE | | |  |  |  |
|  |  | | |  | |  |
| BnFT.A02 | BnaA02T0157300WE | | | FT | | AT1G65480 |
| BnFT.A07 | BnaA07T0277700WE | | |  |  |  |
| BnFT.C02 | BnaC02T0193500WE | | |  |  |  |
| BnFT.C06 | BnaC06T0346000WE | | |  |  |  |
|  |  | | |  | |  |
| BnFUL.A02 | BnaA02T0386000WE | | | FUL | | AT5G60910 |
| BnFUL.A03 | BnaA03T0414900WE | | |  |  |  |
| BnFUL.A09 | BnaA09T0065700WE | | |  |  |  |
| BnFUL.C02 | BnaC02T0504500WE | | |  |  |  |
| BnFUL.C07 | BnaC07T0371300WE | | |  |  |  |
| BnFUL.C09 | BnaC09T0073800WE | | |  |  |  |
|  |  | | |  | |  |
| BnGA20OX3.A02 | BnaA02T0025100WE | | | GA20OX3 | | AT5G07200 |
| BnGA20OX3.A03 | BnaA03T0034500WE | | |  |  |  |
| BnGA20OX3.A10 | BnaA10T0254900WE | | |  |  |  |
| BnGA20OX3.C02 | BnaC02T0021200WE | | |  |  |  |
| BnGA20OX3.C09 | BnaC09T0547000WE | | |  |  |  |
| BnGA20OX3.nn | Bnascaffold1678T0000500WE | | |  |  |  |
|  |  | | |  | |  |
| BnLFY.A06 | BnaA06T0265700WE | | | LFY | | AT5G61850 |
| BnLFY.C02 | BnaC02T0508300WE | | |  |  |  |
| BnLFY.C03 | BnaC03T0486100WE | | |  |  |  |
| **Gene name Westar** | | **Transcript ID**  **Westar** | **Gene name Arabidopsis** | | **Gene ID Arabidopsis** | |
|  | |  |  | |  | |
| BnHBI1.A06 | | BnaA06T0345400WE | HBI1 | | AT2G18300 | |
| BnHBI1.A07 | | BnaA07T0019900WE |  |  |  |  |
| BnHBI1.A09 | | BnaA09T0106800WE |  |  |  |  |
| BnHBI1.C01 | | BnaC01T0134300WE |  |  |  |  |
| BnHBI1.C03 | | BnaC03T0437600WE |  |  |  |  |
| BnHBI1.C09 | | BnaC09T0126900WE |  |  |  |  |
|  | |  |  | |  | |
| BnSEP1.A03 | | BnaA03T0083000WE | SEP1 | | AT5G15800 | |
| BnSEP1.A10 | | BnaA10T0202700WE |  |  |  |  |
| BnSEP1.C03 | | BnaC03T0019400WE |  |  |  |  |
| BnSEP1.C09 | | BnaC09T0478400WE |  |  |  |  |
|  | |  |  | |  | |
| BnSEP2.A01 | | BnaA01T0325800WE | SEP2 | | AT3G02310 | |
| BnSEP2.C01 | | BnaC01T0444600WE |  |  |  |  |
| BnSEP2.C05a | | BnaC05T0555900WE |  |  |  |  |
| BnSEP2.C05b | | BnaC05T0558800WE |  |  |  |  |
|  | |  |  | |  | |
| BnSEP3.A07 | | BnaA07T0097900WE | SEP3 | | AT1G24260 | |
| BnSEP3.A08 | | BnaA08T0200900WE |  |  |  |  |
| BnSEP3.A09 | | BnaA09T0402300WE |  |  |  |  |
| BnSEP3.C03 | | BnaC03T0551900WE |  |  |  |  |
| BnSEP3.C05 | | BnaC05T0224700WE |  |  |  |  |
| BnSEP3.C07 | | BnaC07T0136100WE |  |  |  |  |
|  | |  |  | |  | |
| BnSMZ.A09 | | BnaA09T0478000WE | SMZ | | AT3G54990 | |
| BnSMZ.C08 | | BnaC08T0299400WE |  |  |  |  |
|  | |  |  | |  | |
| BnSOC1.A03 | | BnaA03T0233400WE.1 | SOC1 | | AT2G45660 | |
| BnSOC1.A04 | | BnaA04T0274800WE |  |  |  |  |
| BnSOC1.A05 | | BnaA05T0043700WE |  |  |  |  |
| BnSOC1.C03 | | BnaC03T0197900WE.1 |  |  |  |  |
| BnSOC1.C04a | | BnaC04T0061100WE |  |  |  |  |
| BnSOC1.C04b | | BnaC04T0591600WE |  |  |  |  |
|  | |  |  | |  | |
| BnSPL15.A04 | | BnaA04T0025800WE | SPL15 | | AT3G57920 | |
| BnSPL15.A07 | | BnaA07T0194600WE |  |  |  |  |
| BnSPL15.C04 | | BnaC04T0286900WE |  |  |  |  |
| BnSPL15.C06 | | BnaC06T0236300WE |  |  |  |  |
|  | |  |  | |  | |
| BnTEM1.A08 | | BnaA08T0206500WE | TEM1 | | AT1G25560 | |
| BnTEM1.A09 | | BnaA09T0410100WE |  |  |  |  |
| BnTEM1.C03 | | BnaC03T0544300WE |  |  |  |  |
| BnTEM1.C05 | | BnaC05T0215600WE |  |  |  |  |

**Table S7** Primers used in this study.

| **Primer** | **Sequence** |
| --- | --- |
|  |  |
| Kan2-f | TTGGGTGGAGAGGCTATTCG |
| Kan1-r | CTTCCCGCTTCAGTGACAAC |
| BvWUS_F | CTTGTGGACCAGGATGACCT |
| BvWUS_R | GAGAAGCGTCCGTTCTCAAC |
| W_A03_InDel_F | GGGTTCGAATCCAGGGGTTT |
| W_A03_InDel_R | GAATCGACCAAAATTCAACGTTC |
| W_A03a_F | GGGTTTCTTGTCAATCTGTGC |
| W_A03a_1_R | ACCGGATCAAGCGCAAGG |
| W_A03a_2_R | TGTGATTTTATGGAACCCTCTAAG |
| NGS E2 A02/C02 F | GGTAGATTTAAGAAAACTCAGCC |
| NGS E2 A02/C02 R | TTAAAACTGCATCAGGACTAATTC |
| NGS E3/4 A02/C02 F | TATCCCTTCTCCGTGGAC |
| NGS E3/4 A02/C02 R | ATTAAATATAAGCGCTCTGAAAGT |
| NGS E2 A03/C03 F | GTTGTGTGAATAATTGATTGTCATG |
| NGS E2 A03/C03 R | CTAAGCCTTAGTTACTCCTTTG |
| NGS E4 A03/C03 F | GGGTTTCTTGTCAATCTGTGC |
| NGS E4 A03/C03 R | AACAAGCTTCAACATTAGTTCTG |
| NGS E7 A03/C03 F | CTATGTATTTGAATGGTTGGTATG |
| NGS E7 A03/C03 R | AGATATACAACGTGCACCCTT |
| NGS E2 A03b/C03b F | TAGATTCCAGTGGTGTCTTC |
| NGS E2 A03b/C03b R | AAATAGGAATGATACACGCAGT |
| NGS E4 A03b/C03b F | TACTAGAACTTGTGGAAAGGTTA |
| NGS E4 A03b/C03b R | CAACAAGCTTTAACATTAGTTCTG |
| NGS E2 C09/A10 F | TTCATTCTTTCAAGGGTTAGCTG |
| NGS E2 C09/A10 R | ATGTGTACCGTTAAGTGCGG |
| NGS E4 C09/A10 F | CGTCAGTCAAAAGCTTTGGAC |
| NGS E4 C09/A10 R | TGCTAGCTATAAACTTGGAAGC |
| NGS E7 C09/A10 F | GAGAAGAGTAATCTTGTGCGAG |
| NGS E7 C09/A10 R | TAGTTTTACACCCGAAGCCTC |
